# TWIST1 DNA methylation is a cell marker of airway and parenchymal lung fibroblasts that are differentially methylated in asthma

**DOI:** 10.1186/s13148-020-00931-4

**Published:** 2020-10-02

**Authors:** Rachel L. Clifford, Chen Xi Yang, Nick Fishbane, Jamie Patel, Julia L. MacIsaac, Lisa M. McEwen, Sean T. May, Marcos Castellanos-Uribe, Parameswaran Nair, Ma’en Obeidat, Michael S. Kobor, Alan J. Knox, Tillie-Louise Hackett

**Affiliations:** 1 Nottingham NIHR Biomedical Research Centre, Nottingham MRC Molecular Pathology Node, Division of Respiratory Medicine, University of Nottingham, Nottingham University Hospitals NHS Trust, City Hospital, Nottingham, UK; 2 Centre for Heart Lung Innovation, University of British Columbia, Vancouver, Canada; 3 BC Children’s Hospital Research Institute, Department of Medical Genetics, University of British Columbia, Vancouver, British Columbia Canada; 4 Nottingham Arabidopsis Stock Centre, Plant Sciences Building, School of Biosciences, University of Nottingham, Sutton Bonington Campus, Loughborough, LE12 5RD UK; 5 Firestone Institute for Respiratory Health, St Joseph’s Healthcare and Department of Medicine, McMaster University, Hamilton, Ontario Canada; 6 Department of Anaesthesiology, Pharmacology & Therapeutics, University of British Columbia, Vancouver, Canada

**Keywords:** DNA methylation, Gene expression, Cell marker, Lung, Fibroblast, Airway, Parenchyma, Asthma

## Abstract

**Background:**

Mesenchymal fibroblasts are ubiquitous cells that maintain the extracellular matrix of organs. Within the lung, airway and parenchymal fibroblasts are crucial for lung development and are altered with disease, but it has been difficult to understand their roles due to the lack of distinct molecular markers. We studied genome-wide DNA methylation and gene expression in airway and parenchymal lung fibroblasts from healthy and asthmatic donors, to identify a robust cell marker and to determine if these cells are molecularly distinct in asthma.

**Results:**

Airway (*N* = 8) and parenchymal (*N* = 15) lung fibroblasts from healthy individuals differed in the expression of 158 genes, and DNA methylation of 3936 CpGs (Bonferroni adjusted *p* value < 0.05). Differential DNA methylation between cell types was associated with differential expression of 42 genes, but no single DNA methylation CpG feature (location, effect size, number) defined the interaction. Replication of gene expression and DNA methylation in a second cohort identified TWIST1 gene expression, DNA methylation and protein expression as a cell marker of airway and parenchymal lung fibroblasts, with DNA methylation having 100% predictive discriminatory power. DNA methylation was differentially altered in parenchymal (112 regions) and airway fibroblasts (17 regions) with asthmatic status, with no overlap between regions.

**Conclusions:**

Differential methylation of TWIST1 is a robust cell marker of airway and parenchymal lung fibroblasts. Airway and parenchymal fibroblast DNA methylation are differentially altered in individuals with asthma, and the role of both cell types should be considered in the pathogenesis of asthma.

## Background

Fibroblasts are mesenchymal cells found in the stroma of many tissues and organs throughout the body that are essential for the secretion and maintenance of the extracellular matrix (ECM), in the settings of tissue homeostasis, repair and disease. Fibroblasts are traditionally defined by their spindle-shaped morphology and the relatively non-specific expression of vimentin and S100a4 (Fibroblast specific protein (FSP)-1). However, a lack of lineage markers has hindered the understanding of the specific origins and functions of different fibroblast populations [1]. Gene expression profiling of fibroblasts taken from 35 different anatomic locations, including the skin, lung, aorta, liver, skeletal muscle and prostate, has shown fibroblasts from different organs are molecularly distinct from each other [1, 2]. However, the segregation of fibroblasts by anatomical organ relied on panels of large numbers of genes [2] with few genes distinct to fibroblasts from different organs [1, 2]. Thus, a single gene expression marker of fibroblast anatomical location has been elusive. The problem is more complex in organs such as the lung that contain discreet tissues such as the conducting airways and parenchymal tissue, with distinct fibroblast populations in each structure. Airway and parenchymal fibroblasts have been shown to be morphologically [3, 4] and molecularly distinct [5]. Such variation in fibroblast biology is important to understand, as many common lung diseases such as asthma present with fibrosis in airway versus parenchymal tissues [6, 7], highlighting a need to understand the role of these distinct fibroblast populations.

DNA methylation is commonly studied in the context of disease dysfunction; however, the understanding of its role in regulating normal tissue-specific genome function has only gained momentum in recent years. A vast amount of DNA methylation data now exists across a wide variety of specific cell types, with several studies identifying differentially methylated regions that are both tissue- and organ-specific [8–11].Further, given the stable nature of DNA methylation relative to gene expression, it may provide better molecular markers to distinguish specific tissues and cell types.

**Fig. 1:**
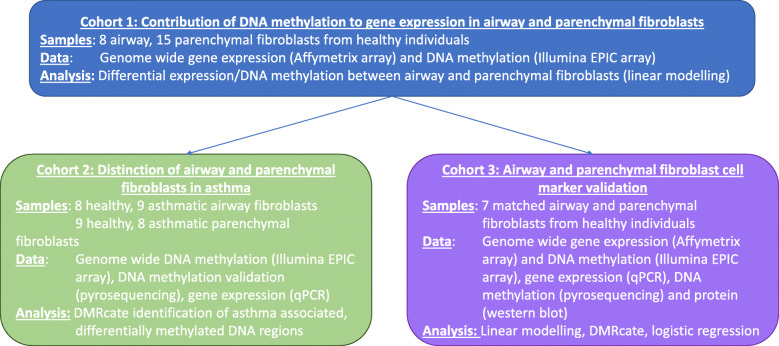
Experimental design summary. Outline of the purpose of the analyses, samples used and analysis undertaken for each section

In the present study (Fig. 1), genome-wide DNA methylation and gene expression were profiled in airway and parenchymal fibroblasts from healthy individuals, to understand if DNA methylation contributes to the heterogeneity of lung fibroblasts and can be used a robust cell marker. Lastly, we assessed whether DNA methylation is altered in airway and parenchymal fibroblasts isolated from individuals with asthma.

## Results

### Airway and parenchymal fibroblasts exhibit different gene expression profiles

We compared genome-wide gene expression between airway fibroblasts (*n* = 8) and parenchymal fibroblasts (*n* = 15) collected from healthy individuals with no respiratory disease or medication history. The demographics of the 23 healthy subjects are provided in Table 1.

**Table 1:** Airway vs parenchymal independent cohort donor demographics

	Airway Fibroblasts	Parenchymal fibroblasts	*p* value
Total N	8	15	
Female/male	6/2	8/7	0.3998
Age (mean ± SEM)	66 ± 3.25	66 ± 2.73	0.787
Smoker (current/ex/non)	0/7/1	4/9/2	0.2588

**Fig. 2:**
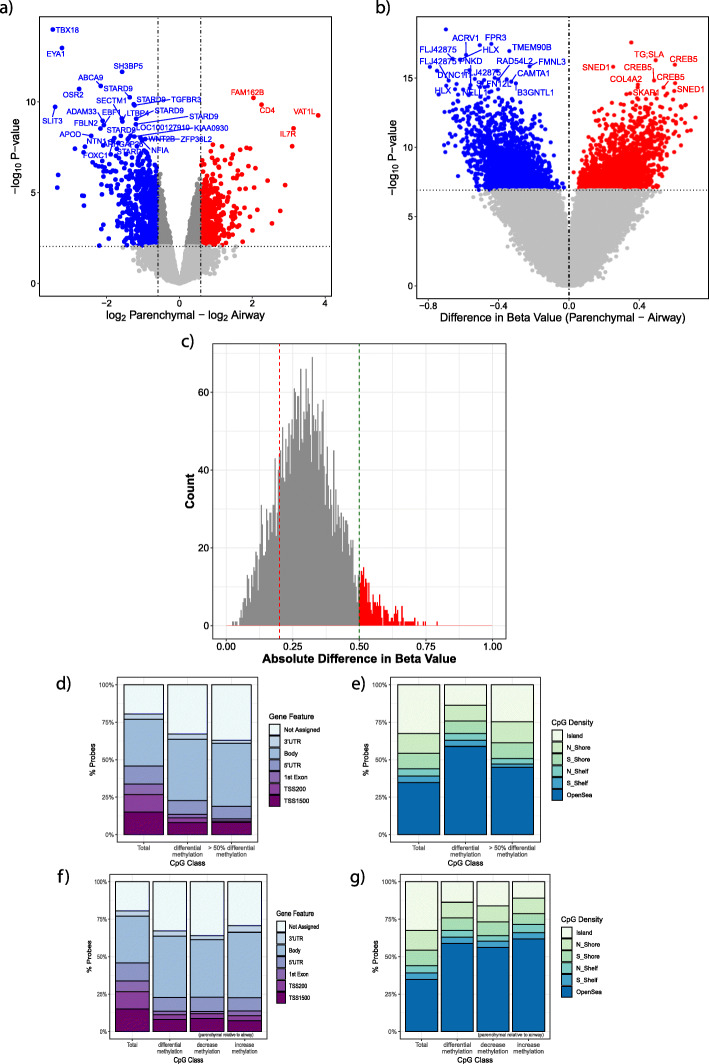
Gene expression and DNA methylation differs between airway and parenchymal fibroblasts. Airway fibroblasts were used as reference. **a** Plot of 53,617 gene expression probes used in the analysis. Red/Blue = Benjamini-Hochberg FDR < 0.05. The horizontal dotted line indicates the *p* value corresponding to Benjamini-Hochberg FDR < 0.05. Red = greater expression in parenchymal fibroblasts, blue = greater expression in airway fibroblasts. **b** Plot of 414,592 CpG probes used in the analysis. Red/Blue = Bonferroni adjusted *p* value < 0.05. Airway fibroblasts were used as reference. Red = greater methylation in parenchymal fibroblasts, blue = greater methylation in airway fibroblasts. The horizontal dotted line indicates the p-value corresponding to adjusted *p* value 0.05. **c** Distribution of DNA methylation (beta) difference between airway and parenchymal fibroblasts in significant probes (Bonferroni adjusted *p* value < 0.05). Red line = difference in beta of 0.2, which approximates a 20% change, green line = beta difference of 0.5. Bars in red indicate an absolute beta difference greater than 0.5 between airway and lung fibroblasts (differential methylation > 50%). **d** Gene feature type for all analysis probes, all significant probes, significant probes with a difference in methylation greater than 50%. **e** CpG density for all analysis probes, all significant probes, significant probes with a difference in methylation greater than 50%. **f** Gene feature type for all analysis probes, all significant probes, significant probes with a decrease in methylation of greater than 50% and significant probes with an increase in methylation of greater than 50%. **g** CpG density type for all analysis probes, all significant probes, significant probes with a decrease in methylation of greater than 50% and significant probes with an increase in methylation of greater than 50%. UTR, untranslated region; TSS, transcription start site; Island, > 200 bp with > 50% GC percentage; Shore, up to 2 kb from a CpG island; N_Shore, North shore upstream of CpG island; S_Shore downstream of CpG island; Shelf, 2–4 kb from a CpG island; OpenSea, regions of the genome without any enrichment of CpG content [12, 13]

We found 3624 probe sets, representing 2619 unique genes, that were significant and differentially expressed between airway and parenchymal lung fibroblasts (Fig. 2a, dark grey points, Benjamini-Hochberg false discovery rate (FDR) < 0.05). Of these, 963 genes had a greater than 1.5-fold difference in gene expression, with 526 genes having higher expression in airway fibroblasts (Fig. 2a, blue points), and 437 genes being more highly expressed in parenchymal fibroblasts (Fig. 2a, red points). Our findings confirm previous observations that normal airway and parenchymal fibroblasts exhibit different gene expression profiles [5]. Of the 963 differentially expressed genes (> 1.5 fold change), only 123 (detailed in Supplemental Table 1) were replicated from the 775 unique genes previously identified as a gene signature of airway and parenchymal fibroblasts [5], demonstrating the transient and variable nature of gene expression.

### Airway and parenchymal fibroblasts exhibit different DNA methylation profiles

Having confirmed that gene expression profiles differed between airway and parenchymal fibroblasts, we next assessed if DNA methylation could provide further molecular distinction of the two cell types. In a site-by-site analysis, DNA methylation identified 3936 CpGs that were significantly differentially methylated between airway and parenchymal lung fibroblasts (Bonferroni adjusted *p* value < 0.05) (Fig. 2b). To understand the characteristics of the CpGs differentially methylated in parenchymal and airway lung fibroblasts, we considered the direction of difference in CpG methylation (Fig. 2b), the magnitude of CpG methylation difference (Fig. 2cc), CpG genomic location (Fig. 2d) and CpG density of the target site location (Fig. 2e). The directional effect of DNA methylation was balanced between the two cell types, with 2088 CpG sites (53%) being more methylated (Fig. 2b, red points) and 1848 (47%) CpG sites being less methylated in parenchymal compared to airway fibroblasts (Fig. 2b, blue points). The effect sizes in terms of DNA methylation differences between airway and parenchymal lung fibroblasts were notable, 3234 (82.16%) CpGs had an absolute beta difference of greater than 0.2 (red line, Fig. 2c) and 240 (6.09%) CpGs had an absolute beta difference of greater than 0.5 (50% methylation difference) (green line, Fig. 2c). Differently methylated CpGs were located across all regions of the genome (Fig. 2d) and within all classifications of CpG density (Fig. 2e); however, they were located primarily in gene body regions (Fig. 2d), and in regions of open sea CpG density (regions of the genome without any enrichment of CpG content [12, 13] (Fig. 2e)). Enrichment within these locations differed significantly from the distribution of the full data set (gene feature, *χ*^2^ *p* value < 0.017 (Fig. 2d); CpG density, *χ*^2^ *p* value < 0.0188, (Fig. 2e)).

To assess whether CpG genomic positioning had any bearing on the magnitude of DNA methylation difference between airway and parenchymal lung fibroblasts, we assessed enrichment to CpG location for sites with a greater than 50% difference in DNA methylation. Enrichment in gene bodies was maintained for sites with a beta difference of > 0.5 (Fig. 2d, *χ*^2^ *p* value < 0.0012); however, enrichment in open sea regions was lost (Fig. 2e, *χ*^2^ *p* value = 0.7124). This suggested that the CpG genomic location of differentially methylated CpGs was not related to effect size but rather sites of smaller effect size contributed significantly to open sea enrichment. To assess whether genomic positioning of a CpG had any influence on the direction of DNA methylation difference between airway and parenchymal fibroblasts, we assessed enrichment of CpG location by direction difference. Similarly, gene body enrichment was maintained regardless of whether a higher (*χ*^2^ *p* value = 0.0256) or lower (*χ*^2^ *p* value = 0.0098) level of DNA methylation was observed in parenchymal fibroblasts relative to airway fibroblasts (Fig. 2f). However, enrichment in open sea regions was observed only for CpGs with a higher level of DNA methylation in parenchymal fibroblasts relative to airway fibroblasts (Fig. 2g; decrease *χ*^2^ *p* value = 0.0621, increase *χ*^2^ *p* value = 0.0022) suggesting some propensity for open sea DNA methylation in parenchymal fibroblasts relative to airway fibroblasts.

Gene set enrichment testing using Gene Ontology (GO), Kyoto Encyclopedia of Genes and Genomes (KEGG) and Reactome identified significant enrichment of CpGs in genes involved in extracellular matrix (ECM) organization, constitution and degradation, cell-cell communication, cell adhesion and muscle contraction (Supplemental Table 2), consistent with previous reports of the different fibrotic functions of fibroblast cell types [3, 4].

### DNA methylation is associated with differential gene expression in airway and parenchymal fibroblasts

**Fig. 3:**
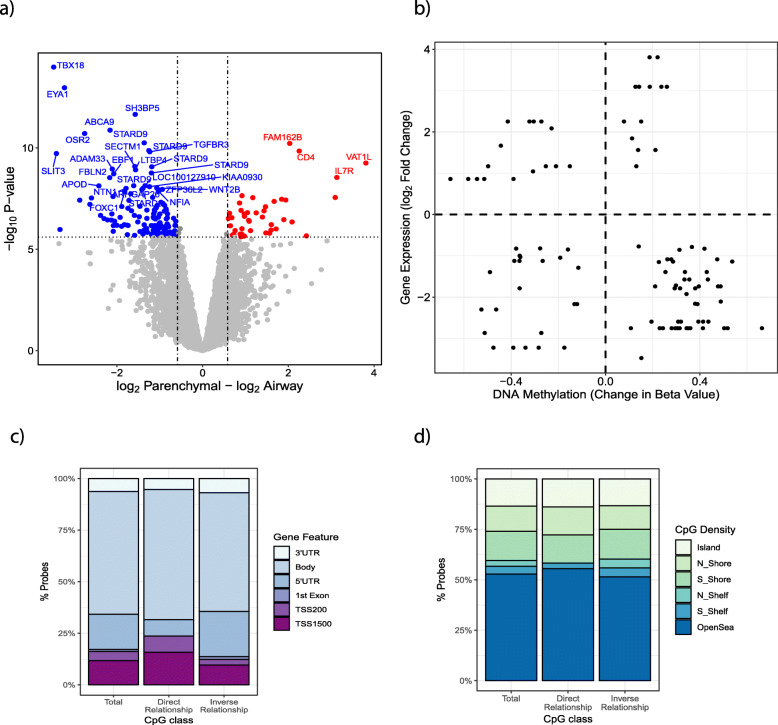
Association between differential DNA methylation and gene expression. **a** Plot of 53,617 probes used in the analysis. Red/Blue = Bonferroni adjusted *p* value < 0.05. Airway fibroblasts were used as reference. The horizontal dotted line indicates the *p* value corresponded to Bonferroni adjusted *p* value 0.05. Red = greater expression in parenchymal fibroblasts, blue = greater expression in airway fibroblasts. **b** Change in DNA methylation plotted against change in gene expression. **c** Gene feature type for all differentially methylated probes associated with a gene that is differentially expressed, differentially methylated probes and differentially expressed genes that display a direct relationship and differentially methylated probes and differentially expressed genes that display an inverse relationship. **d** CpG Density for all differentially methylated probes associated with a gene that is differentially expressed, differentially methylated probes and differentially expressed genes that display a direct relationship and differentially methylated probes and differentially expressed genes that display an inverse relationship

To better understand the relationship between differential gene expression and DNA methylation, we integrated the two datasets. To be consistent with the DNA methylation data, we used Bonferroni-corrected gene expression data, which resulted in 178 probe sets, representing 158 unique genes that were differentially expressed between airway and parenchymal lung fibroblasts (Bonferroni adjusted *p* value < 0.05) (Fig. 3a, dark grey points). Of these, 156 probes had a 1.5-fold difference in gene expression between airway and parenchymal fibroblasts, with 111 genes being more highly expressed in airway fibroblasts (Fig. 3a, blue points) and 45 more highly expressed in parenchymal fibroblasts (Fig. 3a, red points).

Between airway and parenchymal fibroblasts, 42 genes showed differential gene expression associated with differential methylation of at least one DNA methylation probe (104 CpG probes total) (gene names and CpG sites are provided in Supplemental Table 3). Gene set enrichment analysis of the 42 genes identified significant enrichment (enrichment score 1.7) for extracellular matrix constituents (Supplemental Table 4), suggesting DNA methylation may differentially underlie fundamental matrix deposition function in these two cell types. We found both direct and inverse relationships between differences in DNA methylation and fold changes in gene expression (Fig. 3b). Of the 104 differentially DNA methylated CpGs associated with differential gene expression, 68 probes had an inverse relationship, while 36 showed a direct relationship. The direction of the relationship was not associated with gene feature positioning (inverse relationship *χ*^2^ *p* value = 0.9287, direct relationship *χ*^2^ *p* value < 0.3005) (Fig. 3c) or CpG density (inverse relationship *χ*^2^ *p* value = 0.1769, direct relationship *χ*^2^ *p* value < 0.0610) (Fig. 3d).

**Fig. 4:**
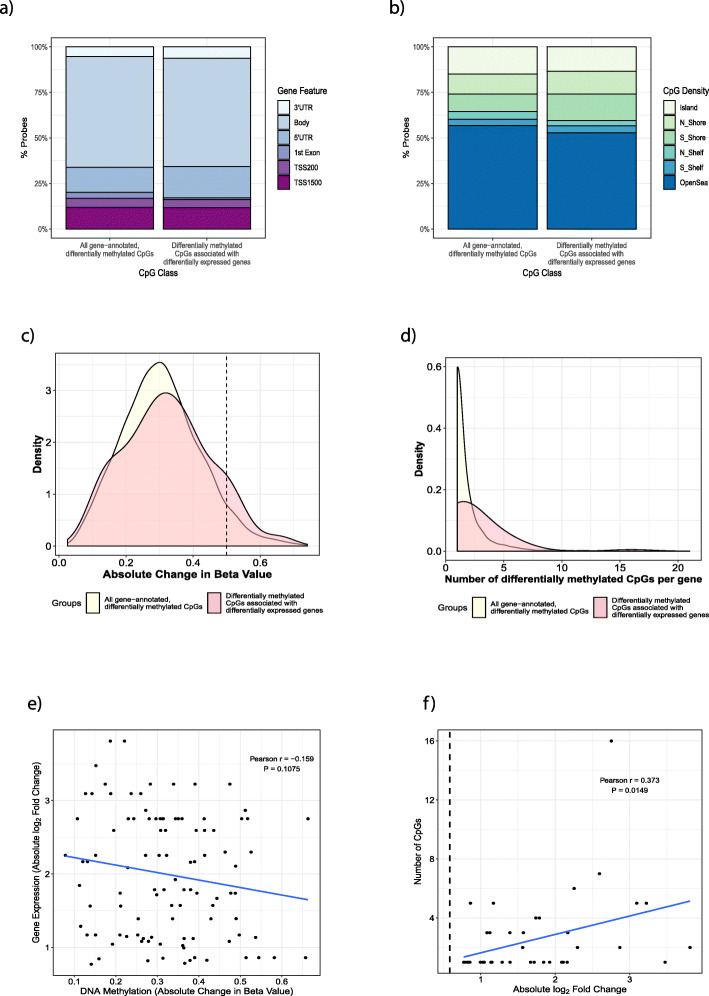
Relationship between differential DNA methylation and gene expression. **a** Gene feature type for all differentially methylated probes and differently methylated probes associated with a gene that is differentially expressed. **b** CpG Density for all differentially methylated probes and differently methylated probes associated with a gene that is differentially expressed. **c** Distribution of CpG probe methylation difference in all differentially methylated probes (yellow) and differently methylated probes associated with a gene that is differentially expressed (pink). **d** Distribution of the number of CpG probes associated with all gene annotated, differentially methylated CpGs (Yellow) and of the number of differently methylated probes associated with a gene that is differentially expressed (pink). **e** Correlation of gene expression and associated probe averaged DNA methylation changes. **f** Correlation of gene expression with number of differentially methylated CpG probes associated with the gene

Genomic location, CpG density, delta beta (DNA methylation difference) values and the number of differentially methylated CpG sites associated with a particular gene are often used to infer biological relevance to DNA methylation data in the absence of gene expression data. To test whether these CpG methylation factors influenced gene expression differences, we compared all differentially methylated CpGs (3936 CpGs) that were annotated to a gene, to the differentially methylated CpGs associated with the 158 genes differentially expressed between airway and parenchymal fibroblasts. We did not find any enrichment for genomic location (Fig. 4a, *χ*^2^ *p* value = 0.4442), density level (Fig. 4b, *χ*^2^ *p* value = 0.9235), delta beta (Fig. 4c, Kolmogorov–Smirnov p = 0.351) or number of differentially methylated CpGs (Fig. 4d, Kolmogorov–Smirnov *p* = 0.137). Specifically, for the 42 genes differentially expressed between airway and parenchymal fibroblasts which mapped to a CpG site with differential DNA methylation, we showed that despite DNA methylation beta values ranging from 7.8 to 50% and being associated with a range of < 1–2.25 log2 fold differences in gene expression, there was no correlation (Fig. 4e, *p* = 0.1075). However, there was a statistically significant but weak correlation, between the number of differentially methylated CpG sites associated with a gene and the extent of differential gene expression (Fig. 4f, Spearman *r* = 0.457, *p* value = 0.00233). These data highlight that DNA methylation should be understood in association with gene expression rather than as a proxy, but that the number of differentially methylated CpGs potentially infers more relevance to gene expression effect size than CpG genomic location, delta beta or density.

### Airway and parenchymal fibroblast DNA methylation profiles are differentially associated with asthmatic status

Having further identified molecular distinction between airway and parenchymal fibroblasts, we investigated the disease relevance of this distinction by assessing whether fibroblast DNA methylation profiles differed with asthmatic status, and importantly whether modifications were shared or distinct between airway and parenchymal fibroblasts. The demographics of the individuals from which cells were isolated are shown in Table 2.

**Table 2:** Asthmatic donors demographics

	Airway fibroblasts			Parenchymal fibroblasts		
	Non-asthmatic	Asthmatic	P value	Non-asthmatic	Asthmatic	***p*** value
**Total** ***N***	8	9		9	8	
**Sex (M/F)**	5/3	3/6	0.6193	7/2	3/5	0.1534
**Age** **(mean ± SEM)**	33.5 ± 8.166	17.67 ± 2.95	0.0754	27.11 ± 5.978	18.75 ± 3.06	0.2566
**Smoking status (non/ex/current)**	4/2/2	7/0/2	0.2505	5/1/3	6/0/2	0.5389
**Bronchodilator (Y/N)**	NA	6/3		N/A	5/3	
**Inhaled steroid (Y/N)**	N/A	1/8		N/A	1/7	
**Oral steroid**	N/A	1/8		N/A	1/7	
**Fatal asthma (Y/N)**	N/A	6/3		N/A	8/3	

**Fig. 5:**
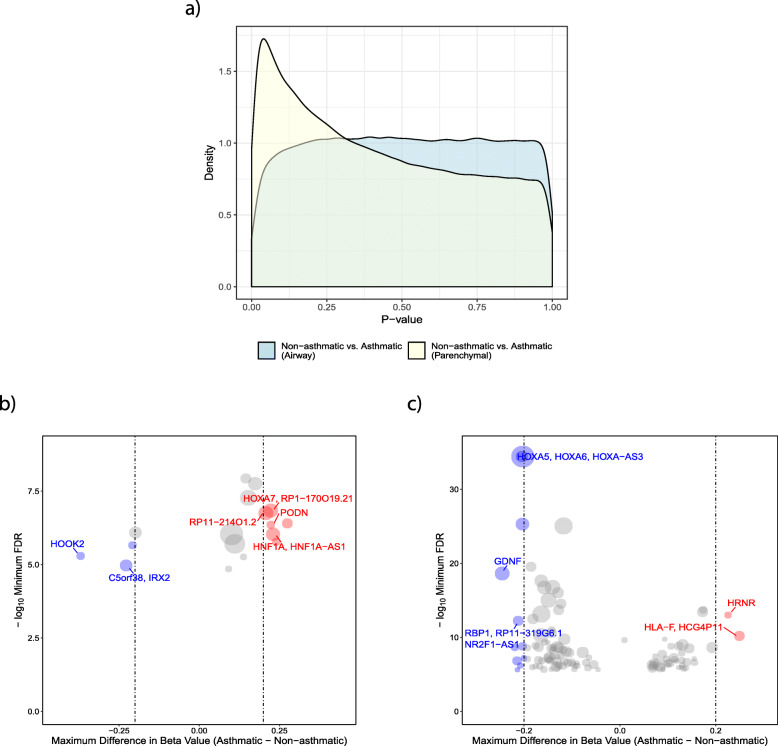
Differential DNA methylation between airway/parenchymal fibroblasts isolated from individuals with/without asthma. **a** *p* value distribution for the association of DNA methylation with asthmatic status in airway (blue) and parenchymal (yellow) fibroblasts. **b** Summary of regional DNA methylation differences between DNA isolated from airway fibroblasts isolated from donors with and without asthma. **c** Summary of regional DNA methylation differences between DNA isolated from parenchymal fibroblasts isolated from donors with and without asthma

**Fig. 6:**
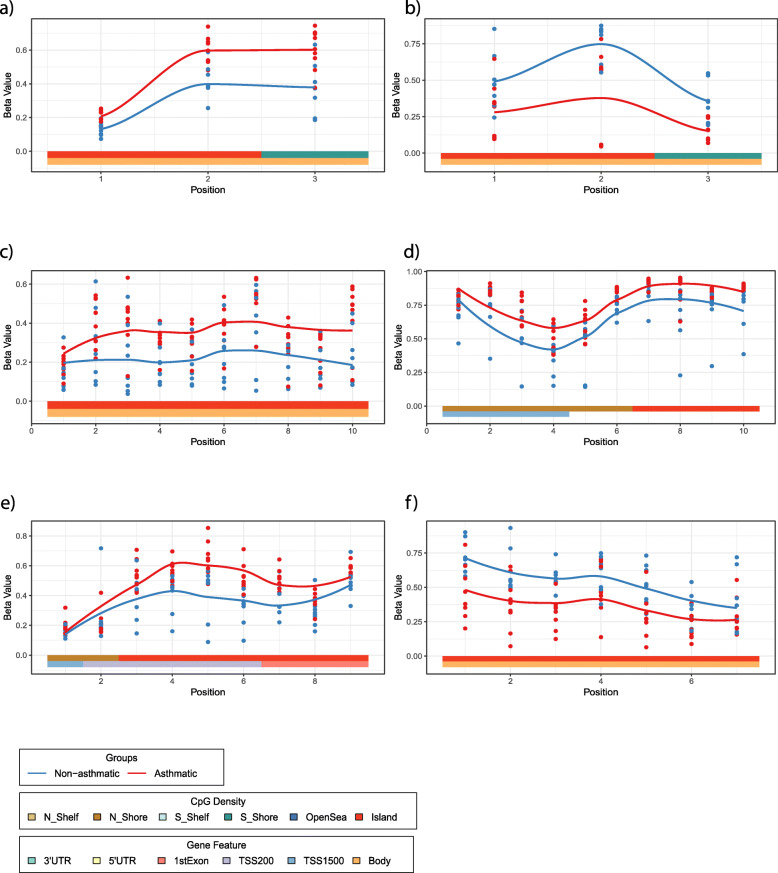
Differential regional DNA methylation in airway fibroblasts isolated from individuals with/without asthma. **a**–**f** Detailed plots of the six regions with a maximum difference in DNA methylation of greater than 20% in airway fibroblasts isolated from individuals with and without asthma. **a** PODN, **b** HOOK2, **c** RP11-214012.2, **d** HOXA7, **e** HNF1A/HNF1A-AS1, **f** C5orf38/IRX2

**Fig. 7:**
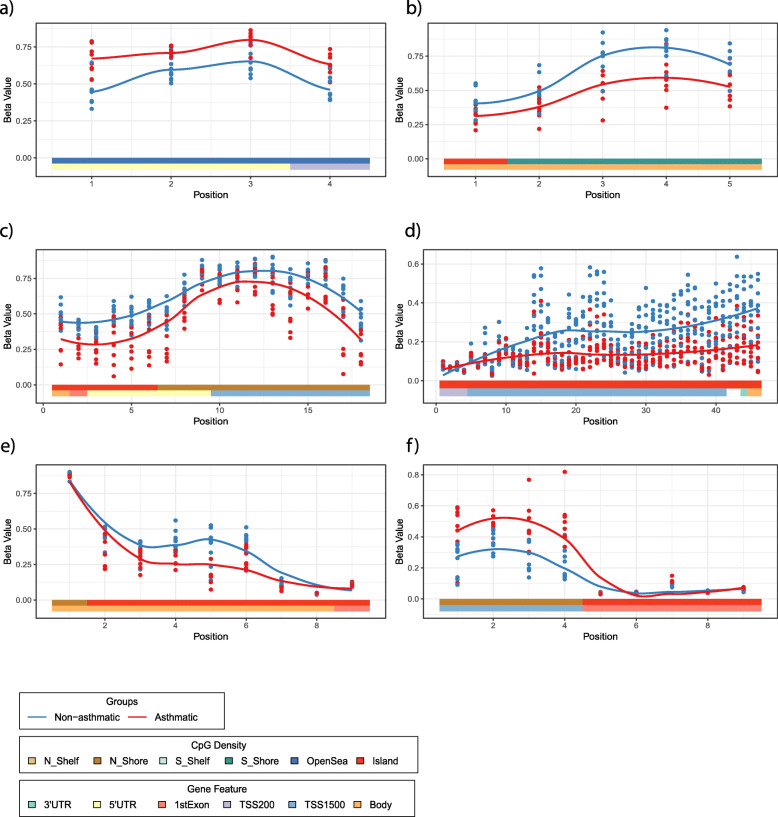
Differential regional DNA methylation in parenchymal fibroblasts isolated from individuals with/without asthma. **a**–**f** Detailed plots of the six regions with a maximum difference in DNA methylation of greater than 20% in parenchymal fibroblasts isolated from individuals with and without asthma. **a** HRNR, **b** NR2F1-AS1, **c** GDNF, **d** HOXA5/A6/A-AS3, **e** RBP1, **f** HLA-F

To test whether asthma was associated with differential methylation, we performed a linear regression and did not observe any statistical differences in DNA methylation at individual CpG sites between asthma and non-asthma cases in either airway or parenchymal lung fibroblasts (Benjamini-Hochberg FDR < 0.05). However, a deviation of the raw *p* value distribution from the null hypothesis suggested an association between asthmatic status and DNA methylation in parenchymal fibroblasts (Fig. 5a, Kolmogorov–Smirnov *p* = 2.2e−16), but not airway fibroblasts (Fig. 5a, Kolmogorov–Smirnov *p* = 0.999). To further investigate this, we looked at aggregated sites via a regional analysis using R package DMRcate. This identified 17 genomic regions differentially methylated in association with asthmatic status in airway fibroblasts (Fig. 5b and details in Supplemental Table 5), and 112 regions in parenchymal fibroblasts (Fig. 5c and details in Supplemental Table 6), with no overlap between the two cell types. The absence of overlap between asthma-associated DNA methylation differences between airway and parenchymal fibroblasts highlights the necessity for them to be investigated independently in lung disease studies, rather than considering a single lung fibroblast population. In airway fibroblasts, 15 of the 17 regions contained three or more CpG sites, of which, 11 were annotated to a known gene promoter (reference genome hg19). Six had a maximum difference in DNA methylation (i.e. at least one probe displayed a mean difference in methylation) of greater than 20% (Δ*β* = 0.2) and were associated with the following genes: *PODN*, *HOOK2, RP11-214O1.2, HOXA7, HNF1A/HNF1A-AS1* and *C5orf38/IRX2* (Fig. 6a–f respectively). In parenchymal fibroblasts, 99 of the 112 regions contained three or more CpG sites, of which 71 were annotated to a known gene. Six of these had a maximum difference in DNA methylation of 20% and were associated with the following genes: *HRNR, NR2F1-AS1, GDNF, HOXA5/6/HOX-AS3, RBP1* and *HLA-F* (Fig. 7a–f respectively).

**Fig. 8:**
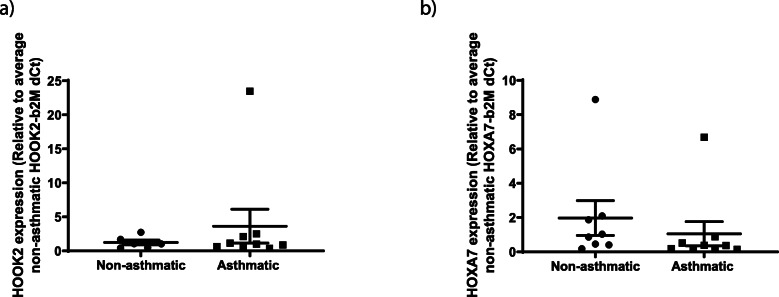
Expression of the genes associated with maximal differential DNA methylation in airway fibroblasts. Gene expression measured by qPCR of **a** HOOK2 and **b** HOXA7 in airway fibroblasts isolated from individuals with and without asthma

**Fig. 9:**
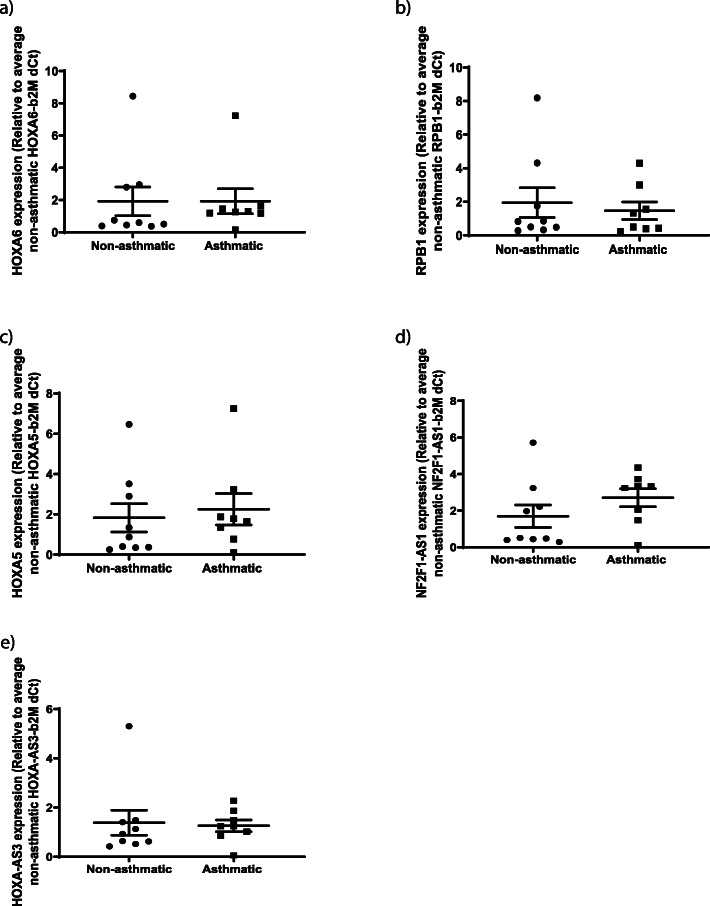
Expression of the genes associated with maximal differential DNA methylation in parenchymal fibroblasts. Gene expression measured by qPCR of **a** HOXA6, **b** RPB1, **c** HOXA5, **d** NF2F1-AS1 and **e** HOXA-AS3 in parenchymal fibroblasts isolated from individuals with and without asthma

Gene expression of the six airway and six parenchymal fibroblast regions specified above identified no expression of *PODN, HNF1A/HNF1A-AS1, C5orf38, IRX2*, *HLA-F, HRNR* or *GDNF* in either fibroblast type*.* For the remaining genes (airway fibroblasts: *HOOK2* and *HOXA7* (Fig. 8a and b); parenchymal fibroblasts: *HOXA5, HOXA6, HOXA-AS3, RB1* and *NR2F1-AS1* (Fig. 9a–e)), there was no differential gene expression between donors with and without asthma.

### Validation of TWIST DNA methylation as a molecular marker of airway and parenchymal fibroblasts

**Fig. 10:**
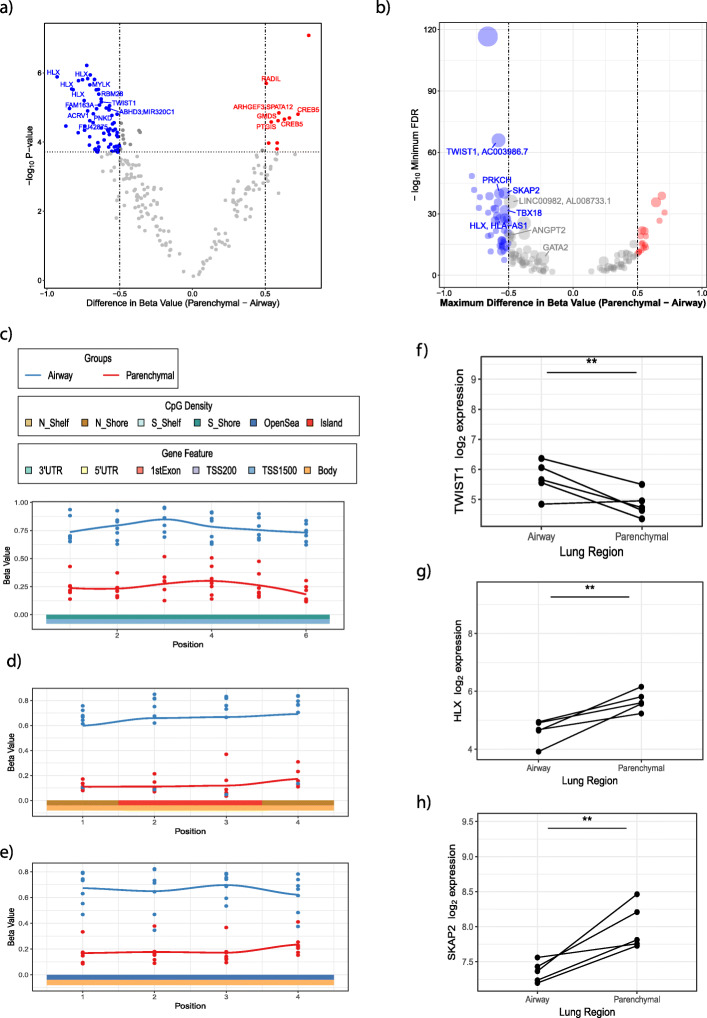
Validation of differential methylation between airway and parenchymal fibroblasts and associated gene expression. **a** Plot of 240 CpG probes used in the analysis. Red/Blue = Bonferroni adjusted *p* value < 0.05. Red = greater expression in parenchymal fibroblasts, blue = greater expression in airway fibroblasts. **b** Summary of regional DNA methylation differences within the 240 CpG probes used in the analysis, between DNA isolated from airway versus parenchymal fibroblasts. **c** Regional beta value plots for TWIST1 in paired samples. **d** Regional beta value plot for HLX in paired samples. **e** Regional beta value plot for SKAP2 in paired samples. Microarray generated gene expression in paired airway and parenchymal fibroblasts for **f** TWIST1, **g** HLX and **h** SKAP2 (**limma moderated *t* test *p* < 0.01)

**Fig. 11:**
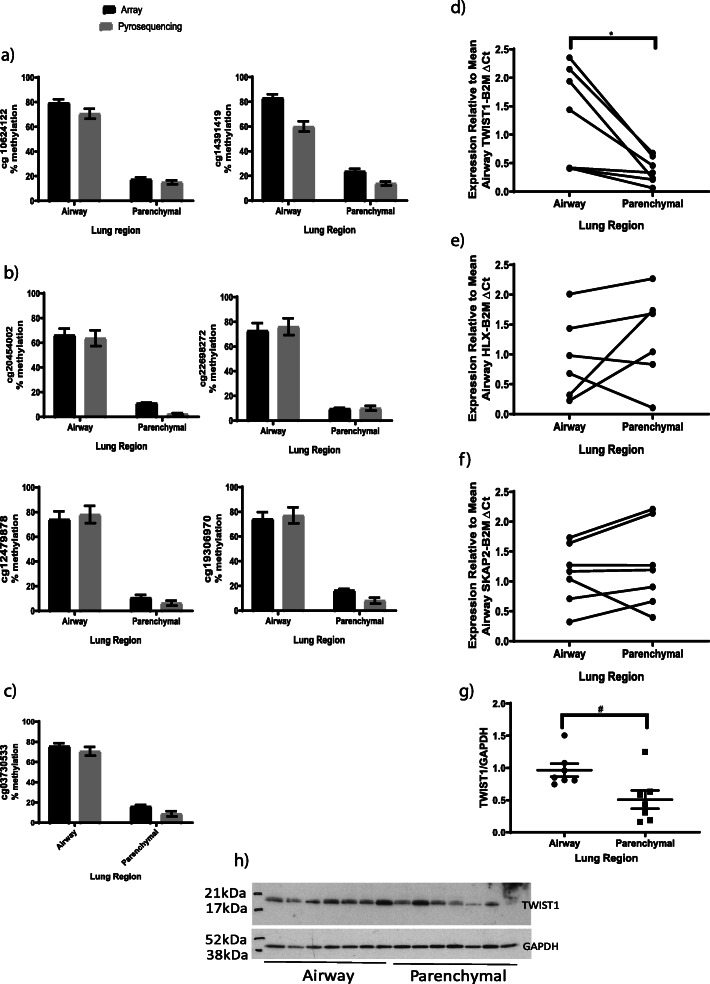
Validation of high confidence targets. Pyrosequencing versus array percent methylation for CpGs identified in regional CpG methylation analyses for **a** TWIST1, **b** HLX and **c** SKAP2. QPCR generated gene expression in the paired samples for **d** TWIST1, **e** HLX, **f** SKAP2 (*Wilcoxon signed-rank test *p* < 0.05). **g** Densitometry and **h** associated western blot for TWIST1 whole cell protein expression (^#^*p* < 0.05 by two-sample *t* test, *n* = 7 airway, *n* = 7 parenchymal)

To investigate if DNA methylation could be used as a molecular cell-type marker, a validation cohort from seven donors with matched airway and parenchymal fibroblasts were used to remove any bias in DNA methylation levels due to inter-individual genetics and environmental exposures. Donors had a mean age of 23.42 (SEM ± 7.5), three were female, two current smokers and five ex-smokers. Eighty-eight of the 240 CpGs previously identified passed Bonferroni correction (*p* < 0.05), and 78 of these exhibited an absolute beta difference of > 0.5 (Fig. 10a). Details of these 78 CpGs are given in Supplemental Table 7. Of the significant CpGs, 67 with a beta difference of > 0.5 were less methylated in parenchymal fibroblasts compared to airway fibroblasts, while 11 CpGs were more methylated (Fig. 10a). To identify a marker with maximum signal-to-noise ratio, specificity and sensitivity, we utilized ‘DMRcate’, a Bioconductor R package [14], to identify de novo differentially methylated regions with CpGs in close genomic proximity. Fourteen regions containing three or more probes were identified as differentially methylated between airway and parenchymal fibroblasts (Fig. 10b and Supplemental Table 8), of which eight were annotated to a gene, allowing for assessment of both gene expression and DNA methylation as a distinguishing marker. This was further restricted to regions containing a CpG identified in the site-by-site analysis by linear modelling, which provided gene regions for further assessment: *TWIST1* (Fig. 10c), *HLX* (Fig. 10d) and *SKAP2* (Fig. 10e). Affymetrix array data identified differential gene expression of *TWIST1* (Fig. 10f), *HLX* (Fig. 10 g) and *SKAP2* (Fig. 10h) between airway and parenchymal fibroblasts, providing three high confidence targets for differentiating between airway and parenchymal fibroblasts. Pyrosequencing verified differential DNA methylation of *TWIST1* (Fig. 11a), *HLX* (Fig. 11b) and *SKAP2* (Fig. 11c) between airway and parenchymal fibroblasts; however, qPCR only validated differential gene expression of *TWIST1* (paired samples Fig. 11d–f, full sample set Supplemental Fig. 1). Further, TWIST1 protein was differentially expressed (higher expression in airway fibroblasts, *p* < 0.05 two sample *t* test), strengthening the evidence for TWIST1 as a DNA methylation cell-type marker capable of distinguishing airway and parenchymal fibroblasts (Fig. 11g/h).

**Fig. 12:**
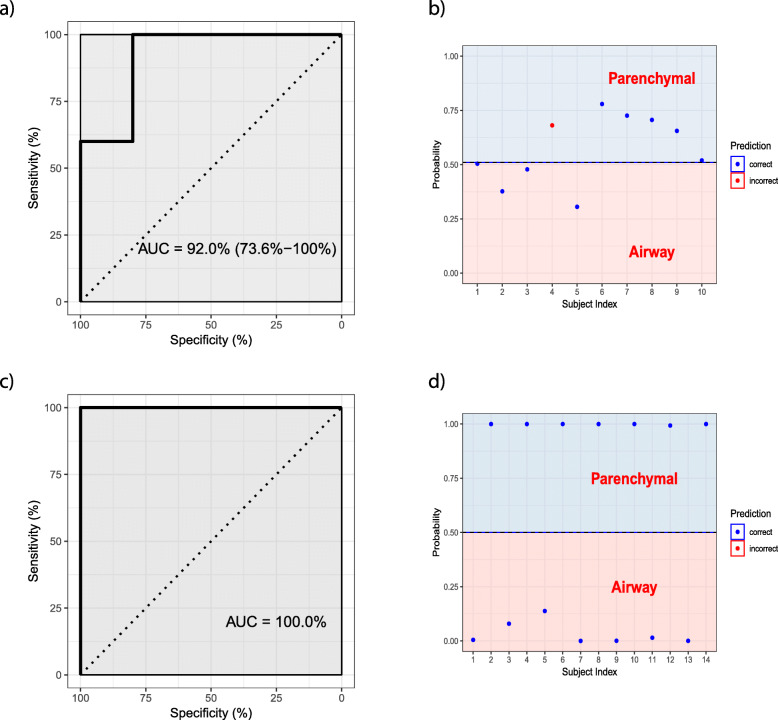
DNA methylation better distinguishes airway and parenchymal fibroblasts that gene expression. **a** Cross-validation AUC for TWIST1 gene expression in the training set. **b** Prediction accuracy of TWIST1 gene expression in the validation set. **c** Cross-validation AUC for six CpGs of TWIST1 in the training set. **d** Prediction accuracy of the six TWIST1 CpGs in the validation set

Finally, logistic regression showed gene expression of TWIST1 identified airway fibroblasts from parenchymal fibroblasts (Fig. 12a) at a validation area under the receiver operating characteristic curve of 92% (95% confidence interval 73.7–100%) (Fig. 12b), while elastic net regularized logistic regression showed DNA methylation of the six TWIST1 CpGs fully distinguished airway fibroblasts from parenchymal fibroblasts (Fig. 12c) at a validation area under the receiver operating characteristic curve of 100% (Fig. 12d). This further suggested that the DNA methylation of CpG sites associated with the TWIST1 gene can be utilized as a molecular marker to distinguish airway and parenchymal fibroblasts for future research.

## Discussion

In the current study, we report that DNA methylation can be utilized as a cellular marker to distinguish airway and parenchymal lung fibroblasts using TWIST-1; further, that DNA methylation profiles can identify differences between airway and parenchymal fibroblasts from asthmatic and non-asthmatic subjects; and lastly, that the relationship between DNA methylation profiles and gene expression signatures is complex.

Airway and parenchymal lung fibroblast transcriptomes have previously been profiled [5], and our data confirmed that the two cell types do indeed have differential gene expression profiles. However, only 123 of the 963 differentially expressed genes in our study (1.5-fold change) replicated the 775 genes previously identified for airway and parenchymal lung fibroblasts by Zhou et al. [5]. One reason could be the use of a more stringent fold change cut off in our analysis (> 1.5 vs > 1.2); however, a sub-analysis of our data showed relaxing our fold-change cut off to that of the previous study still only identified 183 of the previously reported genes. Zhou et al. also combined fibroblasts from asthmatic and non-asthmatic donors in their analysis, having first found no statistical differences in gene expression between the diseased and non-diseased samples. Lastly, technical factors including the cell passage number studied (p4 vs p3 in the Zhou study), the utilization of different microarray platforms, the different normalization methods (Robust Multiarray Averaging (RMA) vs cyclic loss in the Zhou study) or the different statistical tests (limma moderated t-test versus Wilcoxon signed-rank test in the Zhou study) may also account for the discrepancies in our results as compared to the Zhou study. Regardless, these data highlight the transient and variable nature of gene expression which reduces its ability to effectively distinguish between the cell types.

Conversely, DNA methylation is a more stable molecular mark, and tissue-specific differentially methylated regions (tDMRs) have been identified in other tissues [9] suggesting increased potential for utilization as a cellular marker. This study identified 3936 CpGs that were differentially methylated between airway and parenchymal fibroblasts in a cohort of unmatched airway and parenchymal fibroblasts. The airway and parenchymal fibroblast-specific CpGs identified were all enriched within gene bodies and open sea regions. This finding is corroborated by previously identified tissue-specific differentially methylated regions (tDMRs) in blood, saliva, buccal swabs, hair follicles, liver, muscle, pancreas, subcutaneous fat, omentum and spleen that have been shown to be enriched in CpG-poor regions [9]. Interestingly, only 104 of our 3936 (2.6%) differentially methylated CpGs were associated with differential gene expression when using the closest annotated gene name to define the CpG/gene association. Further, the link between gene expression and DNA methylation was not explained by CpG location (gene feature or CpG density), the size of the change in methylation level (delta beta), or the number of differentially methylated CpGs associated with that gene. However, there was a weak correlation between the number of differentially methylated CpGs associated with a gene and the extent of the difference in gene expression. These findings highlight that the relationship between DNA methylation and gene expression differences is complex and need to be validated. As there was no available datasets for us to corroborate our initial finding, we validated the identified 240 high confidence CpGs in a second cohort of matched airway and parenchymal fibroblasts and further confirmed TWIST1 as a cell marker for airway versus parenchymal lung fibroblasts at the DNA methylation, gene expression and protein level. Importantly, elastic net-regularized logistic regression analysis demonstrated that the DNA methylation profile of TWIST1 provided 100% separation between the two cell types indicating that the larger and more defined differences in DNA methylation perform better as a cell-type marker. TWIST1 is a 21 kDa transcription factor [15] that is primarily expressed in mesoderm tissues from the early stages of embryo development and is involved in the specification and differentiation of mesenchyme tissues. It is therefore probable that TWIST1 is a cell marker for airway and parenchymal fibroblasts due to its distinct regulation of mesenchymal cell phenotypes. The lack of an independent validation cohort remains a limitation to the study; however, the cells used in the current study were isolated at three different geographical locations using the same tissue culture protocols, limiting the opportunity for our differences to be due to isolation bias.

Understanding the regulation of genes like TWIST1 by DNA methylation may have important implications for understanding the role of airway and parenchymal fibroblasts in lung health and disease. Differential DNA methylation in asthma has been studied in the airway epithelium [16–19], but not in lung fibroblast populations. We identified 17 airway and 112 parenchymal fibroblast differentially methylated DNA regions that were associated with asthma, with no overlap between the two cell types. None of the airway but two of the parenchymal fibroblast asthma-associated DNA methylation regions were annotated to genes with previously identified genome-wide association study genetic risk loci for asthma [20], TSLP (rs1837253) and GATA3 (rs2589561), with the SNP and the differentially methylated regions being between ~ 0.6 and 1 Mbp apart. Importantly, the differences in the number of asthma-associated differences in DNA methylation observed between airway and parenchymal fibroblasts was independent of fatal and non-fatal asthma, and the therapeutic use of inhaled bronchodilators and steroids as airway and parenchymal fibroblasts were isolated from the same asthmatic individuals. The larger number of perturbations to DNA methylation in association with asthma in parenchymal fibroblasts versus airway fibroblasts was surprising based on the understanding that the parenchyma’s contribution to the asthmatic phenotype is thought to be minimal. However, airway fibrosis is driven by myofibroblasts (an α smooth muscle actin (αSMA) positive fibroblast subtype), and recently an αSMA-positive fibroblast subtype was identified in the lung parenchyma, with approximately three times more αSMA-positive cells in the parenchyma of individuals with asthma compared to non-asthmatic control subjects [21, 22] suggesting that parenchymal-derived fibroblasts in asthmatics may play a role. Furthermore, it has been shown that there is more parenchymal extracellular matrix in asthmatic lungs compared to controls [22]. These studies highlight an emerging role for the parenchyma fibroblast in asthma pathology and the necessity to work with the most appropriate cell type when investigating human disease.

## Conclusions

In conclusion, our study identified in two independent sample cohorts, that genome-wide and targeted DNA methylation profiles of *TWIST1* can distinguish between airway and parenchyma-derived lung fibroblasts. Further, airway and parenchymal fibroblast-related differences in DNA methylation and associated gene expression, indicate these two cells are phenotypically different and likely perform separate and distinct roles in normal lung physiology. Further, we show that airway and parenchymal fibroblast DNA methylation is differentially altered in individuals with asthma and the role of both cell types should be considered in the pathogenesis of asthma.

## Methods

### Isolation and culture of airway and parenchymal fibroblasts

Primary cultures of airway and parenchymal fibroblasts from healthy and asthmatic individuals were isolated from lung biopsies, intrapulmonary airway and parenchymal lung tissue obtained from lung cancer resections (from the normal margin) or non-transplantable donor lungs. Airway and parenchymal fibroblasts were derived using the outgrowth techniques as previously described [3, 23] at three different sites. Briefly, 2 mm^2^ tissue explants were placed in 6-well tissue culture plates with DMEM (Sigma) containing 10% fetal bovine serum (GIBCO, Life Technologies), penicillin (100 U/ml), streptomycin (100 μg/ml) and l-glutamine (4 mM) in a 5% CO_2_-humidified incubator. Media was replaced regularly until cellular outgrowth reached confluence. Tissue pieces were removed and destroyed, and cells were harvested using trypin/EDTA solution (Sigma). All cells for this study were cultured at the same time under the exact same conditions. Samples were generated from cells at passage 4 except for two asthmatic airway fibroblast samples at passage 5, a single healthy airway fibroblasts sample at passage 3, and two asthmatic parenchymal fibroblast samples at passage 5. Cells at the required passage were grown to confluence in 6-well plates and serum starved for 24 h prior to lysis for DNA, RNA and protein isolation. The tissue was obtained and cells extracted with the approval of each of the research ethics boards for each of the academic institutions involved: McMaster University (Hamilton Integrated Research Ethics Board Ref:00-1839), University of British Columbia (Providence Health Care Research Ethics Board Ref:H13-02173) and University of Nottingham (East Midlands Research Ethics Committee Ref: 08/H0407/1).

### DNA and RNA isolation

DNA and RNA were simultaneously isolated from each sample using the AllPrep DNA/RNA Mini Kit (Qiagen) as per the manufacturer’s instructions and assessed for quality and quantity using a NanoDrop^TM^ 8000 Spectrophotometer (Thermo Fisher Scientific).

### Protein isolation

Cells were washed with PBS, lysed in RIPA buffer (Sigma) and stored at − 80 until required.

### Bisulfite conversion and DNA methylation arrays

Seven hundred fifty nanograms of purified genomic DNA was bisulfite converted using the EZ DNA Methylation Kit (Zymo Research) as per the manufacturer’s instructions. Specific incubation conditions for the Illumina Infinium Methylation Assay were used as per the manufacturer’s protocol Appendix. Samples were eluted in 12 μl of the provided elution buffer. Bisulfite-converted DNA was assessed for concentration and quality using a NanoDrop^TM^ 8000 Spectrophotometer (Thermo Fisher Scientific), and 160 ng of the conversion product was used for genome-wide DNA methylation quantification at over 485,000 CpG sites using the Illumina Infinium HumanMethylation450 BeadChip array, according to the manufacturer’s protocols.

### DNA methylation data quality control and normalization

IDAT files produced by GenomeStudio were imported into the R statistical software (version 3.2.1) using the minfi package (v. 1.14.0) [24]. The 65 known quality control SNP probes were used to cluster all samples to detect anomalies within samples from the same donor. Probes were excluded from further analysis according to several criteria: first, 1402 probes were found to have either a detection *p* value < 0.05 in at least 1% of samples or had less than 3 bead count in at least 5% of samples; second, the 65 SNP probes; third, 59,593 probes were found to be cross-hybridized to other parts of the genome [25]; and fourth, 9925 probes on the XY chromosomes. 414,592 probes remained for analysis. Filtered probes were normalized using the funtooNorm algorithm [26], which extends the funNorm procedure [27] and is purported to correct for unwanted variation while preserving important differences in methylation patterns between different cell types. We employed the normalization option of principal components regression with 5 principal components. Two values of DNA methylation were calculated, beta-values (*β-*values) and *M*-values. *β*-values are the ratio of all methylated probe intensities over total signal intensities (methylated and unmethylated) and have a range from 0 to 1. They approximately represent percent methylation. *M*-values are the logit transformation of *β*-values and are more statistically robust [28]. All statistical analyses were performed using *M*-values, while *β*-values were used for visualization and interpretability purposes. Principal components analysis was performed for quality control of the *M*-values. Two replicates between passage 3 and passage 4 of the same donor provided correlation *r*^2^ of 0.9869 and 0.9948, suggesting minimal genome-wide DNA methylation dysfunction due to passage.

### Differential DNA methylation analysis

#### Airway versus parenchymal in healthy individuals

Samples were split into two groups: those used in the initial analysis in which we used all available samples regardless of whether matched samples from airway and parenchyma were available, and a second group, containing only samples from donors from which we obtained both airway and parenchymal samples. The paired samples were not included in the initial analysis and represent a completely independent data set.

Linear regression analysis was applied to the initial group using the *limma* package in R [29] while adjusting for age as a covariate. As large changes in DNA methylation were apparent, we considered only CpG sites to be significant if they had a Bonferroni-adjusted *p* value < 0.05, and an effect size on methylation-β of more than 0.5. With these sites, a similar analysis was done on the paired group using their DNA methylation differences between parenchyma and airway, which is analogous to a paired *t* test, but also adjusted for age. Filtering the latter results to chromosomes with more than one hit, we identified genomic clustering of the CpG sites in differentially methylated regions (DMRs) using the *DMRcate* package in R [14], which uses Gaussian kernel smoothing to find patterns of differential methylation, agnostic to genomic annotation. We used the authors’ recommended bandwidth (*λ*) of 1000 base pairs and scaling factor (C) of 2, though we were using a sparse set of sites, the intention was to find regions with large effect sizes. Gene set testing was performed with the methylglm function of methylGSA [30] in R, which adjusts for the number of CpGs associated with a gene.

#### Asthma versus non-asthma

Airway and parenchymal fibroblasts were considered separately. Linear regression analysis using the *limma* package in R was used to identify individually differentially methylated CpG sites. Regional differences in DNA methylation associated with asthmatic status were identified using the *DMRcate* package in R including all CpGs specified by a nominal linear modelling *p* value limit of < 0.001.

### Gene expression microarray analysis

Whole-genome transcriptome analysis was conducted by hybridizing samples of total RNA to Affymetrix Human Gene 2.1 ST Arrays Strips (Affymetrix, Santa Clara, CA, USA). A minimum RIN score of 8 was used as cut off for inclusion in the microarray analysis. Two hundred fifty-nanogram RNA was used for all samples. All steps were conducted at the Nottingham Arabidopsis Stock Centre.

### Differential gene expression analysis

Raw CEL files were read into R, RMA background corrected with quantile normalization and log2 transformed using the Oligo package [31–34]. Linear regression analysis was applied to sample groups using the *limma* package in R to identify differentially expressed genes [29]. Both Benjamini-Hochberg and Bonferroni**-**corrected significance levels are reported. Integration with DNA methylation was done with Bonferroni-corrected data for both DNA methylation (3936 CpGs) and gene expression (158 genes), using Affymetrix gene annotation for the gene expression data and Illumina Closest Transcription Start Site annotation for DNA methylation data.

### Bisulfite PCR-pyrosequencing

DNA methylation data were confirmed using Pyrosequencing at 7 CpG sites present in both the DMRcate and linear modelling data comparing airway and parenchymal fibroblasts: TWIST1, cg10624122, cg14391419; HLX, cg20454002, cg22698272, cg12479878, cg19306970; SKAP2, cg03730533. We were unable to design a functional assay for SKAP2 cg12140851. Bisulfite PCR-pyrosequencing assays were designed with PyroMark Assay Design 2.0 (Qiagen). The regions of interest were amplified by PCR using the HotstarTaq DNA polymerase kit (Qiagen) as follows: 15 min at 95 °C (to activate the Taq polymerase), 45 cycles of 95 °C for 30 s, 58 °C for 30 s, and 72 °C for 30 s, and a 5-min 72 °C extension step. For pyrosequencing, single-stranded DNA was prepared from the PCR product with the Pyromark™ Vacuum Prep Workstation (Qiagen) and sequencing was performed using sequencing primers on a Pyromark™ Q24 pyrosequencer (Qiagen). The quantitative levels of methylation for each CpG dinucleotide were calculated with Pyromark Q24 software (Qiagen). Primer sequences are shown in Table 3.

**Table 3:** Pyrosequencing primer details

Target	Forward primer	Reverse primer	Sequencing primer	Assay position
TWIST cg10624122	AGTATATAGTGTTGGGGTGGG	*AGTATATAGTGTTGGGGTGGG	AGGTTAGTTTATGGGTTTTTTAG	5
TWIST1 cg14391419	AGTATATAGTGTTGGGGTGGG	*ACCTTACTCCAACCCAAAAA	TTGGGGTGGGGGTAG	1
HLX cg20454002	ATGGAAGTGAGGGATATATAGGAATTTT	*TCTCTCTCCCAACAATTACCAA	AGGGATATATAGGAATTTTT	2
HLX cg22698272	*GGTGTATTTTTTAGGTTTGTAGT	CCCCTTAAACAATACTCTAAAATTTTTCC	ACAAAAACCTCCTTAATAAAATCT	1
HLX cg12479878	TTTTAGGATTGAAGTTTTTAGGGTTGTT	*CTACCCCTTTTCAAAAAAAACCA	GGTGTAGTAATTTTATAATTGGG	1
HLX cg19306970	AGTTTATGTTTGGGTGTTTGGATAT	*CCCTTAAACCTAAATAATAACAACC	TTTGGGTGTTTGGATATA	1
SKAP1 cg03730533	GTAGTAATTTGATAATAAGAAAAGGTTAGT	*ACCTCCCACCCTTCTCTCCC	TTTAGTTAGGTTTTTAGAATTTTTT	1

*Biotinylated

### Reverse transcription and qPCR

0.5 μg of RNA was reverse transcribed using SuperScript IV (Invitrogen) as per the manufacturer’s instructions. The resulting 20 μl cDNA samples were diluted to a total volume of 200 μl using nuclease-free water. cDNA was amplified using PerfeCTa SYBR Green FastMix (Quanta bio), with 2 μl template and 200 nM primers in a 10-μl reaction using a Stratagene Mx3000P/3005P system. Thermal cycler conditions included incubation at 95 °C for 10 min, followed by 40 cycles of 95 °C for 10 s, 60 °C for 30 s, and 72 °C for 20 s. Data was collected in MxPro, a single product was confirmed by melt curve analysis and Ct values were exported to excel for analysis. Expression was expressed by the ΔΔCt method relative to β_2_-Microglobulin (β_2_M) Ct and mean airway fibroblast target/β_2_M ΔCt. Primer sequences are as follows: β_2_-Microglobulin, forward 5′-AATCCAAATGCGGCATCT-3′, reverse 5′-GAGTATGCCTGCCGTGTG-3′; TWIST1, forward 5′-GCCCGGAGACCTAGATGTCATT-3′, reverse 5′-CCCACGCCCTGTTTCTTTGA-3′; HLX, forward 5′-CGCTGAGAGATCTCACTTCCC-3′, reverse 5′-TCAGGATTGCAGAAGCCTCG-3′; SKAP2, forward 5′-GTTCTTAATCCGGGCCGCTA-3′, reverse 5′-TCAACATCTGCCAACAGGTTC-3′. Primer utility was tested on reference cDNA (Takara) for positive amplification prior to assessment of samples.

### Western blotting

Fifteen-microgram whole cell lysate protein samples were subject to electrophoresis in 15% SDS-polyacrylamide gel. Separated proteins were electroblotted to polyvinylidene difluoride membranes, and the blot was blocked for 1 h at room temperature with blocking buffer (0.1% TBST with 5% fat-free dried milk powder). The blot was then incubated with 1:1000 dilution TWIST 1 (clone MAB6230, R&D Systems) or 1:10,000 dilution GAPDH (Abcam) at 4 °C overnight. The blot was washed with 0.1% TBST and incubated with HRP-conjugated anti-mouse secondary Abs (DakoCytomation, Cambridge, U.K.) (1:2000 dilution with 5% fat-free dried milk in 0.1% TBST) for 1 hour. The blot was washed again and then incubated with Clarity western ECL substrate (Bio-Rad). The densitometry analysis was performed in ImageJ.

### Classification using elastic net-regularized logistic regression

Elastic net-regularized logistic regression was applied to both DNA methylation data (microarray) and gene expression data (microarray) to distinguish airway fibroblasts from parenchymal fibroblasts. Data were split into a training set and a validation set. Samples from the initial non-matched groups were used as the training set while samples from the paired groups were used as the validation set. Elastic net performs both shrinkage of regression coefficients and feature selection to identify features (genes or CpG sites) that are highly discriminative with respect to the phenotypes (fibroblast type). The mixing percentage (*α*) and regularization shrinkage parameter (*λ*) were tuned at different values to build biomarker panels of various sizes. Area under the receiver operating characteristic curve (AUC) obtained from leave-one-out cross-validation (LOOCV) of the training set was used as the evaluation metric to identify the best-performing panel. The optimal cut-off for predicted probabilities was determined using Youden’s index method to maximize both sensitivity and specificity. The validation set was subsequently tested through the best-performing panel to calculate the validation AUC and prediction accuracy. All the analyses were performed using the *glmnet* package in R [35].

## Supplementary information

**Additional file 1.** QPCR generated gene expression of paired airway and parenchymal fibroblasts for TWIST1, HLX and SKAP2.

**Additional file 2.** Supplementary Tables 1-8

## Data Availability

The dataset generated and analysed during the current study is available in the Gene Expression Omnibus (GEO) repository, https://www.ncbi.nlm.nih.gov/geo/ GSE157651.

## Abbreviations

ECM: Extracellular matrix
FSP-1: Fibroblast-specific protein 1
TGFβ1: Transforming growth factor beta 1
SEM: Standard error of the mean
FDR: False discovery rate
GO: Gene Ontology
KEGG: Kyoto Encyclopedia of Genes and Genomes
