# Supplementary figures and images for: TWIST1 DNA methylation is a cell marker of airway and parenchymal lung fibroblasts that are differentially methylated in asthma
Source: Clin Epigenetics. 2020 Oct 2;12:145. doi: 10.1186/s13148-020-00931-4 (PMC7531162; doi:10.1186/s13148-020-00931-4)

## Slide 1
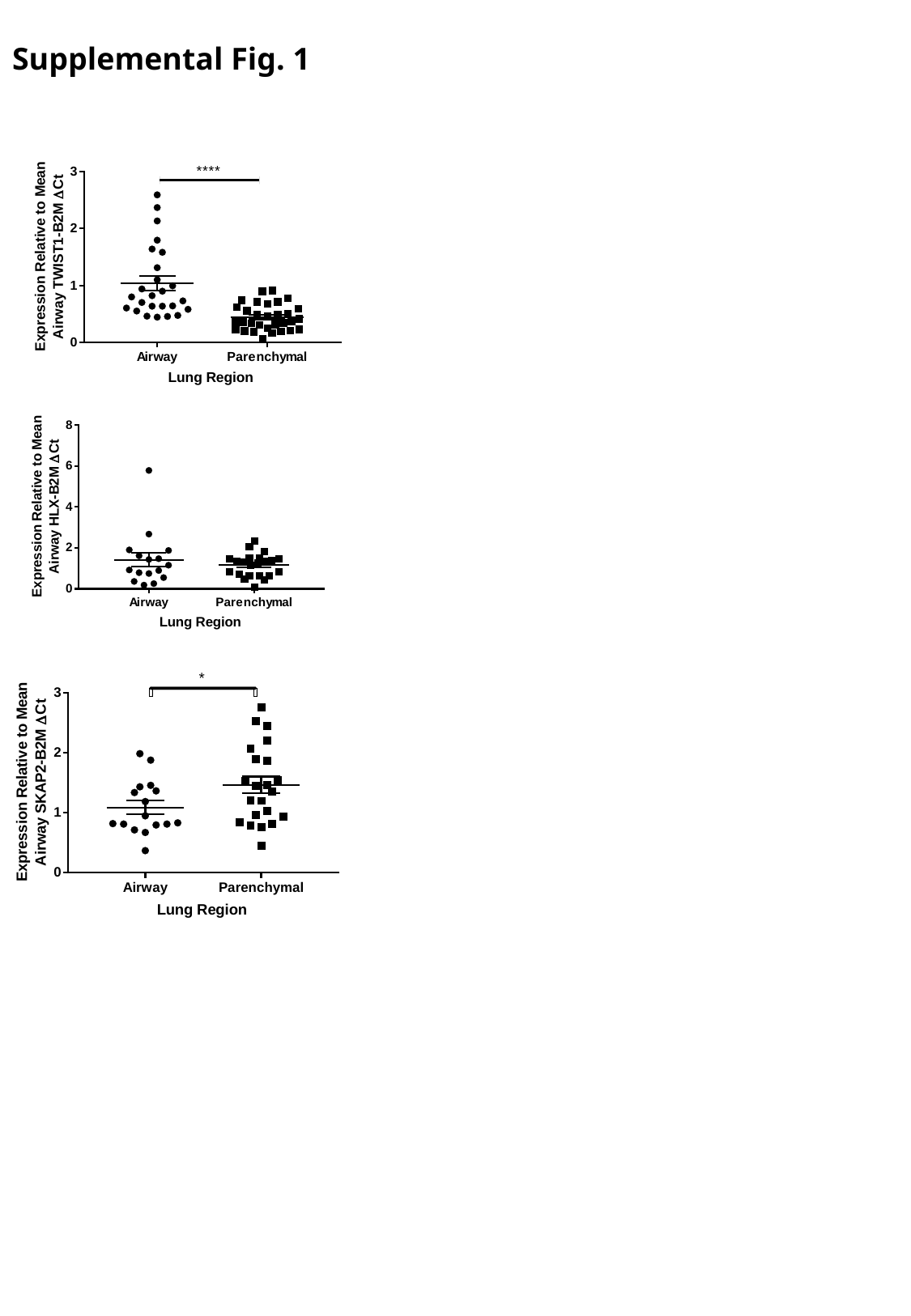

# Supplemental Fig. 1

Supplement: Supplementary file 1 — Additional file 1. QPCR generated gene expression of paired airway and parenchymal fibroblasts for TWIST1, HLX and SKAP2. [file 13148_2020_931_MOESM1_ESM.zip › Supplemental Figure 1.pptx]
