# Supplementary material for: TWIST1 DNA methylation is a cell marker of airway and parenchymal lung fibroblasts that are differentially methylated in asthma
Source: Clin Epigenetics. 2020 Oct 2;12:145. doi: 10.1186/s13148-020-00931-4 (PMC7531162; doi:10.1186/s13148-020-00931-4)
Supplement: Supplementary file 2 — Additional file 2. Supplementary Tables 1-8 [file 13148_2020_931_MOESM2_ESM.zip › ST1.docx]

**Supplementary Table 1: Summary of 123 genes differentially expressed between airway and parenchymal fibroblasts in both our data set and GSE27335**

| Gene | Probe | Fold Change | FDR | Probe_in_GSE27335 | Fold Change in GSE27335 |
| --- | --- | --- | --- | --- | --- |
| A4GALT | 16935690 | 1.548 | 0.00165 | A_23_P57570 | 1.913 |
| ABCA5 | 16848318 | 2.001 | 0.00475 | A_23_P78018 | 1.34 |
| ABCA6 | 16848219 | 2.200 | 0.00007 | A_23_P500400 | 2.014 |
| ACPP | 16945682 | 3.038 | 0.00012 | A_24_P37589 | 1.504 |
| ACVRL1 | 16751381 | 1.771 | 0.03570 | A_24_P945113 | 4.959 |
| AHNAK2 | 16797196 | 1.814 | 0.01690 | A_24_P178618 | 3.058 |
| AKR1C3 | 16702007 | 1.560 | 0.00893 | A_23_P138541 | 2.204 |
| ANKRD29 | 16854301 | 5.212 | 0.00006 | A_23_P412577 | 2.789 |
| APBB1IP | 16703407 | 2.597 | 0.00322 | A_23_P401700 | 7.882 |
| APOBEC3G | 16930199 | 1.529 | 0.00590 | A_24_P942933 | 1.405 |
| ARHGAP23 | 17121496 | 1.797 | 0.01239 | A_24_P920207 | 1.647 |
| ARHGAP26 | 16990483 | 2.627 | 0.00001 | A_24_P28165 | 1.963 |
| BICC1 | 16705089 | 1.516 | 0.00144 | A_23_P86532 | 1.456 |
| BMP6 | 17004657 | 1.668 | 0.01833 | A_23_P19624 | 1.612 |
| C3 | 16867784 | 3.836 | 0.02519 | A_23_P101407 | 1.455 |
| C5orf38 | 16982775 | 1.867 | 0.00034 | A_23_P331235 | 2.743 |
| CAMK1D | 17118754 | 2.076 | 0.00127 | A_23_P124252 | 1.436 |
| CASP6 | 16978959 | 1.876 | 0.00066 | A_23_P500799 | 1.279 |
| CD34 | 16698801 | 2.323 | 0.00044 | A_32_P220798 | 2.261 |
| CD55 | 16676764 | 1.689 | 0.00054 | A_23_P374862 | 1.873 |
| CDH13 | 16821377 | 8.541 | 0.00002 | A_32_P85999 | 10.7 |
| CLCN4 | 17101422 | 2.633 | 0.00039 | A_23_P217741 | 1.982 |
| CLDN1 | 16962661 | 3.331 | 0.03330 | A_23_P57784 | 5.517 |
| COLEC12 | 16853399 | 2.649 | 0.00202 | A_23_P27306 | 5.207 |
| CRLF1 | 16870443 | 4.920 | 0.00007 | A_23_P56197 | 12.87 |
| CSF1 | 16668420 | 1.706 | 0.00289 | A_23_P45133 | 1.528 |
| CSRP1 | 16697938 | 1.663 | 0.00058 | A_24_P225448 | 2.578 |
| DDO | 17022553 | 2.251 | 0.00002 | A_23_P30603 | 2.381 |
| DENND5B | 16762941 | 1.676 | 0.00882 | A_24_P541919 | 1.367 |
| DES | 16891261 | 1.805 | 0.03530 | A_23_P90710 | 5.242 |
| DNAJC22 | 16750792 | 1.965 | 0.00278 | A_32_P407245 | 2.292 |
| DNMBP | 16717412 | 1.743 | 0.01982 | A_23_P328206 | 1.606 |
| DTL | 16677201 | 1.633 | 0.03569 | A_23_P10385 | 1.486 |
| ELF4 | 17114038 | 1.529 | 0.01594 | A_24_P340066 | 1.261 |
| ELOVL6 | 16978995 | 1.565 | 0.01316 | A_23_P7361 | 1.878 |
| EPHA4 | 16908897 | 1.880 | 0.01752 | A_24_P274219 | 1.819 |
| EPHB2 | 16660596 | 2.219 | 0.00017 | A_23_P200067 | 2.034 |
| EPHB3 | 16949062 | 1.877 | 0.00140 | A_23_P95060 | 1.739 |
| EPHX1 | 16678114 | 1.764 | 0.00005 | A_23_P34537 | 1.705 |
| EYA1 | 17078134 | 9.339 | 0.00000 | A_23_P502363 | 3.473 |
| FAM126A | 17055804 | 1.755 | 0.00074 | A_23_P8582 | 1.456 |
| FAM180A | 17063221 | 1.533 | 0.01080 | A_32_P204218 | 1.341 |
| FAM60A | 16762921 | 1.525 | 0.00130 | A_23_P13663 | 1.531 |
| FBLN1 | 16931237 | 2.203 | 0.00013 | A_23_P303113 | 1.608 |
| FBN1 | 16808793 | 2.622 | 0.00023 | A_23_P65678 | 2.166 |
| FGF14 | 16780754 | 1.755 | 0.00752 | A_23_P88033 | 2.275 |
| FGF18 | 16992347 | 2.752 | 0.01824 | A_23_P93027 | 6.972 |
| FMO2 | 16673713 | 7.475 | 0.00037 | A_23_P355295 | 6.89 |
| FOXC1 | 17004208 | 3.527 | 0.00001 | A_32_P205110 | 3.124 |
| FRK | 17022916 | 1.916 | 0.01575 | A_23_P133665 | 1.28 |
| GAS1 | 17095499 | 3.159 | 0.00004 | A_23_P83134 | 2.603 |
| GPX3 | 16991192 | 1.523 | 0.01957 | A_23_P133474 | 4.497 |
| HBEGF | 17000724 | 1.872 | 0.00246 | A_24_P140608 | 2.175 |
| HLA-DMA | 17037303 | 1.531 | 0.01756 | A_24_P50245 | 2.025 |
| HMOX1 | 16929562 | 1.574 | 0.01234 | A_23_P120883 | 2.488 |
| HOXB2 | 16846218 | 1.508 | 0.00215 | A_23_P107283 | 1.469 |
| HOXB5 | 16846254 | 1.936 | 0.00213 | A_23_P363316 | 1.932 |
| HOXB6 | 16846259 | 1.890 | 0.00001 | A_24_P933151 | 1.869 |
| HSBP1 | 16821398 | 1.594 | 0.00181 | A_23_P328511 | 1.261 |
| HSD11B1 | 16676988 | 2.970 | 0.00209 | A_23_P63209 | 3.203 |
| HSPA2 | 16785379 | 2.285 | 0.00171 | A_23_P88303 | 3.904 |
| ISLR | 16802960 | 1.935 | 0.00121 | A_23_P3312 | 6.727 |
| ISYNA1 | 16870401 | 1.712 | 0.00378 | A_23_P5131 | 1.645 |
| JUP | 16844872 | 3.590 | 0.00002 | A_23_P501822 | 7.898 |
| KIAA0513 | 16821562 | 1.724 | 0.00818 | A_23_P206310 | 1.945 |
| KIF20A | 16989636 | 2.300 | 0.00295 | A_23_P256956 | 1.517 |
| LIFR | 16995500 | 1.992 | 0.00051 | A_24_P397386 | 2.124 |
| LXN | 16960911 | 2.112 | 0.00951 | A_23_P6674 | 1.892 |
| LZTS1 | 17075221 | 1.600 | 0.02248 | A_23_P20443 | 2.475 |
| MARVELD2 | 16985688 | 2.078 | 0.00074 | A_23_P401675 | 2.403 |
| MFAP5 | 16760953 | 1.974 | 0.00203 | A_23_P87700 | 27.64 |
| MFSD6 | 16888822 | 2.064 | 0.00005 | A_23_P28530 | 1.703 |
| MT1L | 16819213 | 3.157 | 0.00320 | A_23_P427703 | 2.099 |
| MT1M | 16819224 | 3.522 | 0.00048 | A_23_P66241 | 2.114 |
| MT1X | 16819264 | 3.838 | 0.00002 | A_23_P303242 | 1.966 |
| MYH11 | 16824258 | 5.817 | 0.00733 | A_24_P70183 | 12.78 |
| NES | 16694689 | 1.783 | 0.00276 | A_23_P103672 | 3.458 |
| NPR3 | 16983765 | 3.139 | 0.00694 | A_23_P253536 | 2.449 |
| PARP3 | 16941278 | 1.642 | 0.01169 | A_24_P402779 | 1.343 |
| PDE4DIP | 17120056 | 2.068 | 0.00072 | A_32_P110550 | 1.534 |
| PDE7A | 17077774 | 1.616 | 0.00697 | A_23_P123478 | 1.602 |
| PLA2G4A | 16675197 | 2.498 | 0.01686 | A_23_P11685 | 2.342 |
| PLA2R1 | 16904152 | 3.003 | 0.00025 | A_23_P142830 | 2.552 |
| PPA1 | 16715133 | 1.606 | 0.00346 | A_23_P161338 | 1.333 |
| PPP1R14A | 16871915 | 2.764 | 0.00003 | A_24_P296772 | 23.09 |
| PXK | 16942202 | 1.600 | 0.00140 | A_32_P115606 | 1.886 |
| RAPH1 | 16907488 | 2.203 | 0.00005 | A_24_P924862 | 1.647 |
| RASD2 | 16929610 | 4.654 | 0.00008 | A_23_P143673 | 1.249 |
| RECK | 17084838 | 1.682 | 0.00116 | A_23_P83028 | 2.042 |
| RRM2 | 16877019 | 2.158 | 0.00103 | A_24_P225616 | 1.509 |
| S100A4 | 16693449 | 1.995 | 0.00450 | A_23_P94800 | 5.696 |
| SASH1 | 17013520 | 1.898 | 0.00002 | A_23_P93442 | 1.86 |
| SERPINE2 | 16909021 | 1.774 | 0.02693 | A_23_P50919 | 3.568 |
| SERPINF1 | 16829570 | 1.638 | 0.00963 | A_23_P100660 | 3.215 |
| SFT2D2 | 16673477 | 1.851 | 0.00003 | A_23_P148785 | 1.512 |
| SH3BP5 | 16951150 | 2.974 | 0.00000 | A_24_P148750 | 3.3 |
| SLC2A12 | 17023799 | 2.238 | 0.00893 | A_23_P395001 | 1.831 |
| SLC4A4 | 16967631 | 2.206 | 0.00033 | A_32_P349145 | 1.783 |
| SNX29 | 16815925 | 1.708 | 0.00028 | A_23_P367816 | 1.275 |
| SOCS2 | 16755131 | 1.515 | 0.00245 | A_24_P230675 | 2.29 |
| SOX5 | 16762288 | 1.517 | 0.01576 | A_24_P925186 | 1.271 |
| SPOCD1 | 16684581 | 1.756 | 0.00159 | A_23_P431388 | 2.274 |
| SRPX | 17110071 | 1.639 | 0.00148 | A_23_P96383 | 1.735 |
| STARD8 | 17104344 | 1.513 | 0.00761 | A_23_P387630 | 1.887 |
| SYTL2 | 16742963 | 1.839 | 0.00181 | A_23_P53193 | 2.481 |
| TAF9B | 17112339 | 1.751 | 0.00170 | A_24_P391431 | 1.279 |
| TBX18 | 17021323 | 11.118 | 0.00000 | A_23_P134041 | 5.189 |
| TBX19 | 16673489 | 1.631 | 0.00098 | A_23_P137705 | 1.347 |
| TLR4 | 17088527 | 1.667 | 0.00679 | A_32_P66881 | 3.911 |
| TM2D2 | 17076496 | 1.532 | 0.01695 | A_23_P502678 | 1.449 |
| TMEM155 | 16979502 | 3.343 | 0.00003 | A_23_P408195 | 3.106 |
| TMEM30B | 16793644 | 2.570 | 0.00017 | A_32_P129752 | 3.828 |
| TNC | 17097661 | 2.095 | 0.00082 | A_23_P157865 | 8.969 |
| TNFSF10 | 16961616 | 2.391 | 0.03823 | A_23_P121253 | 2.179 |
| TOM1L1 | 16836214 | 1.706 | 0.00078 | A_24_P290709 | 1.411 |
| TOP2A | 16844312 | 2.212 | 0.00331 | A_23_P118834 | 1.691 |
| TSHZ2 | 16914925 | 2.956 | 0.00021 | A_23_P154627 | 2.929 |
| TSPAN2 | 16691314 | 5.354 | 0.00026 | A_24_P62659 | 1.739 |
| UBASH3B | 16732676 | 3.179 | 0.00002 | A_24_P192933 | 1.733 |
| USP18 | 16926942 | 1.655 | 0.02696 | A_23_P132159 | 3.358 |
| XG | 17101214 | 10.040 | 0.00016 | A_23_P72487 | 7.351 |
| ZFHX4 | 17070177 | 2.177 | 0.00004 | A_23_P43095 | 1.658 |
| ZNF627 | 16858509 | 1.606 | 0.03334 | A_23_P135730 | 1.303 |
