# Supplementary material for: TWIST1 DNA methylation is a cell marker of airway and parenchymal lung fibroblasts that are differentially methylated in asthma
Source: Clin Epigenetics. 2020 Oct 2;12:145. doi: 10.1186/s13148-020-00931-4 (PMC7531162; doi:10.1186/s13148-020-00931-4)
Supplement: Supplementary file 2 — Additional file 2. Supplementary Tables 1-8 [file 13148_2020_931_MOESM2_ESM.zip › ST2.docx]

**Supplementary Table 2: Summary of gene set enrichment testing for genes associated with CpGs differentially methylated between airway and parenchymal fibroblasts.**

| **Gene Set Type** | **ID** | **Description** | **Size** | **pvalue** | **padj** |
| --- | --- | --- | --- | --- | --- |
| KEGG | 4060 | Cytokine-cytokine receptor interaction | 265 | 3.45E-06 | 0.000152 |
| KEGG | 4514 | Cell adhesion molecules (CAMs) | 133 | 4.61E-05 | 0.001014 |
| KEGG | 4510 | Focal adhesion | 200 | 0.001458 | 0.021388 |
| KEGG | 4080 | Neuroactive ligand-receptor interaction | 272 | 0.002533 | 0.024989 |
| KEGG | 4270 | Vascular smooth muscle contraction | 116 | 0.00284 | 0.024989 |
| Reactome | R-HSA-1474244 | Homo sapiens: Extracellular matrix organization | 278 | 1.63E-13 | 2.63E-11 |
| Reactome | R-HSA-1474228 | Degradation of the extracellular matrix | 127 | 5.92E-06 | 0.00048 |
| Reactome | R-HSA-416476 | G alpha (q) signalling events | 201 | 1.02E-05 | 0.000548 |
| Reactome | R-HSA-373076 | Class A/1 (Rhodopsin-like receptors) | 285 | 5.5E-05 | 0.002229 |
| Reactome | R-HSA-1500931 | Cell-Cell communication | 116 | 0.000124 | 0.004015 |
| Reactome | R-HSA-397014 | Muscle contraction | 200 | 0.000252 | 0.006795 |
| Reactome | R-HSA-381426 | Regulation of Insulin-like Growth Factor (IGF) transport and uptake by Insulin-like Growth Factor Binding Proteins (IGFBPs) | 114 | 0.000415 | 0.009611 |
| Reactome | R-HSA-500792 | GPCR ligand binding | 409 | 0.000603 | 0.01222 |
| Reactome | R-HSA-418594 | G alpha (i) signalling events | 378 | 0.001206 | 0.021704 |
| Reactome | R-HSA-202733 | Cell surface interactions at the vascular wall | 128 | 0.002979 | 0.048266 |
| GO | GO:0030198 | extracellular matrix organization | 384 | 5.08E-13 | 9.2E-10 |
| GO | GO:0043062 | extracellular structure organization | 472 | 1.51E-12 | 1.37E-09 |
| GO | GO:0005201 | extracellular matrix structural constituent | 248 | 9.97E-12 | 6.02E-09 |
| GO | GO:0045785 | positive regulation of cell adhesion | 455 | 2.41E-08 | 1.09E-05 |
| GO | GO:0060326 | cell chemotaxis | 303 | 5.83E-08 | 2.11E-05 |
| GO | GO:0050921 | positive regulation of chemotaxis | 148 | 9.65E-08 | 2.92E-05 |
| GO | GO:0030595 | leukocyte chemotaxis | 226 | 1.18E-07 | 3.06E-05 |
| GO | GO:0007160 | cell-matrix adhesion | 240 | 1.82E-07 | 4.05E-05 |
| GO | GO:1904018 | positive regulation of vasculature development | 268 | 2.01E-07 | 4.05E-05 |
| GO | GO:0045765 | regulation of angiogenesis | 449 | 2.65E-07 | 4.65E-05 |
| GO | GO:0031589 | cell-substrate adhesion | 373 | 2.82E-07 | 4.65E-05 |
| GO | GO:0005539 | glycosaminoglycan binding | 209 | 5.41E-07 | 8.18E-05 |
| GO | GO:0050920 | regulation of chemotaxis | 212 | 7.9E-07 | 0.00011 |
| GO | GO:0008201 | heparin binding | 151 | 8.57E-07 | 0.000111 |
| GO | GO:0045766 | positive regulation of angiogenesis | 231 | 1.12E-06 | 0.000135 |
| GO | GO:0032103 | positive regulation of response to external stimulus | 357 | 1.21E-06 | 0.000137 |
| GO | GO:0005178 | integrin binding | 128 | 1.88E-06 | 0.000201 |
| GO | GO:0005604 | basement membrane | 103 | 3.51E-06 | 0.000354 |
| GO | GO:0030278 | regulation of ossification | 213 | 4.26E-06 | 0.000406 |
| GO | GO:0070663 | regulation of leukocyte proliferation | 240 | 8.42E-06 | 0.000763 |
| GO | GO:0005126 | cytokine receptor binding | 270 | 1.38E-05 | 0.001174 |
| GO | GO:0002792 | negative regulation of peptide secretion | 146 | 1.42E-05 | 0.001174 |
| GO | GO:0050663 | cytokine secretion | 262 | 1.82E-05 | 0.001365 |
| GO | GO:0097530 | granulocyte migration | 112 | 1.84E-05 | 0.001365 |
| GO | GO:0098858 | actin-based cell projection | 226 | 1.88E-05 | 0.001365 |
| GO | GO:0032944 | regulation of mononuclear cell proliferation | 225 | 1.97E-05 | 0.001372 |
| GO | GO:0001503 | ossification | 448 | 2.12E-05 | 0.001416 |
| GO | GO:0002688 | regulation of leukocyte chemotaxis | 115 | 2.19E-05 | 0.001416 |
| GO | GO:0009897 | external side of plasma membrane | 201 | 2.28E-05 | 0.001423 |
| GO | GO:0030282 | bone mineralization | 120 | 2.52E-05 | 0.001523 |
| GO | GO:0019838 | growth factor binding | 147 | 3.36E-05 | 0.001965 |
| GO | GO:0019955 | cytokine binding | 117 | 3.47E-05 | 0.001965 |
| GO | GO:0031214 | biomineral tissue development | 181 | 4.19E-05 | 0.002304 |
| GO | GO:0050670 | regulation of lymphocyte proliferation | 222 | 4.46E-05 | 0.002379 |
| GO | GO:0043502 | regulation of muscle adaptation | 120 | 4.91E-05 | 0.0025 |
| GO | GO:0097529 | myeloid leukocyte migration | 200 | 4.96E-05 | 0.0025 |
| GO | GO:0050709 | negative regulation of protein secretion | 140 | 5.64E-05 | 0.002762 |
| GO | GO:0002687 | positive regulation of leukocyte migration | 133 | 6.19E-05 | 0.002918 |
| GO | GO:2000146 | negative regulation of cell motility | 381 | 6.28E-05 | 0.002918 |
| GO | GO:0010811 | positive regulation of cell-substrate adhesion | 113 | 6.84E-05 | 0.003099 |
| GO | GO:0042445 | hormone metabolic process | 276 | 8.19E-05 | 0.003623 |
| GO | GO:0002685 | regulation of leukocyte migration | 205 | 8.54E-05 | 0.003687 |
| GO | GO:0003300 | cardiac muscle hypertrophy | 122 | 8.97E-05 | 0.003783 |
| GO | GO:0022407 | regulation of cell-cell adhesion | 476 | 9.76E-05 | 0.004022 |
| GO | GO:0035265 | organ growth | 187 | 0.000116 | 0.004654 |
| GO | GO:0055074 | calcium ion homeostasis | 493 | 0.000126 | 0.004959 |
| GO | GO:0001818 | negative regulation of cytokine production | 327 | 0.00013 | 0.00497 |
| GO | GO:0014897 | striated muscle hypertrophy | 125 | 0.000134 | 0.00497 |
| GO | GO:0030336 | negative regulation of cell migration | 364 | 0.000134 | 0.00497 |
| GO | GO:0050679 | positive regulation of epithelial cell proliferation | 214 | 0.000146 | 0.005282 |
| GO | GO:0007159 | leukocyte cell-cell adhesion | 409 | 0.000163 | 0.005599 |
| GO | GO:0048018 | receptor ligand activity | 480 | 0.000167 | 0.005599 |
| GO | GO:0042116 | macrophage activation | 109 | 0.000167 | 0.005599 |
| GO | GO:0051924 | regulation of calcium ion transport | 299 | 0.000167 | 0.005599 |
| GO | GO:1903039 | positive regulation of leukocyte cell-cell adhesion | 255 | 0.000175 | 0.00576 |
| GO | GO:0050707 | regulation of cytokine secretion | 221 | 0.000192 | 0.006227 |
| GO | GO:0051271 | negative regulation of cellular component movement | 399 | 0.000212 | 0.006647 |
| GO | GO:0010594 | regulation of endothelial cell migration | 268 | 0.000213 | 0.006647 |
| GO | GO:1903037 | regulation of leukocyte cell-cell adhesion | 362 | 0.00023 | 0.007007 |
| GO | GO:0014896 | muscle hypertrophy | 127 | 0.000232 | 0.007007 |
| GO | GO:0090130 | tissue migration | 426 | 0.000242 | 0.007197 |
| GO | GO:0010810 | regulation of cell-substrate adhesion | 215 | 0.000255 | 0.007443 |
| GO | GO:0001952 | regulation of cell-matrix adhesion | 126 | 0.000274 | 0.007874 |
| GO | GO:0010632 | regulation of epithelial cell migration | 340 | 0.000304 | 0.008605 |
| GO | GO:0022409 | positive regulation of cell-cell adhesion | 298 | 0.000317 | 0.008766 |
| GO | GO:0043500 | muscle adaptation | 135 | 0.000321 | 0.008766 |
| GO | GO:0006874 | cellular calcium ion homeostasis | 478 | 0.000327 | 0.008766 |
| GO | GO:0055017 | cardiac muscle tissue growth | 112 | 0.000329 | 0.008766 |
| GO | GO:0007162 | negative regulation of cell adhesion | 304 | 0.000334 | 0.008767 |
| GO | GO:0050673 | epithelial cell proliferation | 481 | 0.00036 | 0.009206 |
| GO | GO:0051480 | regulation of cytosolic calcium ion concentration | 370 | 0.000368 | 0.009206 |
| GO | GO:0090132 | epithelium migration | 420 | 0.00037 | 0.009206 |
| GO | GO:0098857 | membrane microdomain | 326 | 0.000373 | 0.009206 |
| GO | GO:0051668 | localization within membrane | 145 | 0.000376 | 0.009206 |
| GO | GO:0010631 | epithelial cell migration | 417 | 0.000387 | 0.009359 |
| GO | GO:0040013 | negative regulation of locomotion | 414 | 0.000475 | 0.011328 |
| GO | GO:0043542 | endothelial cell migration | 326 | 0.000492 | 0.011586 |
| GO | GO:0070661 | leukocyte proliferation | 303 | 0.000504 | 0.01171 |
| GO | GO:1903825 | organic acid transmembrane transport | 126 | 0.000568 | 0.01287 |
| GO | GO:1905039 | carboxylic acid transmembrane transport | 126 | 0.000568 | 0.01287 |
| GO | GO:0002526 | acute inflammatory response | 239 | 0.000578 | 0.012948 |
| GO | GO:0008360 | regulation of cell shape | 124 | 0.000626 | 0.01383 |
| GO | GO:0051897 | positive regulation of protein kinase B signaling | 184 | 0.000648 | 0.014092 |
| GO | GO:0044449 | contractile fiber part | 224 | 0.000653 | 0.014092 |
| GO | GO:0098589 | membrane region | 337 | 0.000677 | 0.014443 |
| GO | GO:0045121 | membrane raft | 324 | 0.000727 | 0.015332 |
| GO | GO:0014706 | striated muscle tissue development | 424 | 0.000748 | 0.015591 |
| GO | GO:0071219 | cellular response to molecule of bacterial origin | 207 | 0.000765 | 0.015756 |
| GO | GO:0072503 | cellular divalent inorganic cation homeostasis | 500 | 0.000785 | 0.015985 |
| GO | GO:0010959 | regulation of metal ion transport | 466 | 0.000869 | 0.01735 |
| GO | GO:1901343 | negative regulation of vasculature development | 219 | 0.000871 | 0.01735 |
| GO | GO:0060541 | respiratory system development | 206 | 0.000892 | 0.017579 |
| GO | GO:0090287 | regulation of cellular response to growth factor stimulus | 315 | 0.000943 | 0.018376 |
| GO | GO:0060537 | muscle tissue development | 444 | 0.001002 | 0.019322 |
| GO | GO:0006909 | phagocytosis | 341 | 0.001043 | 0.019904 |
| GO | GO:0004896 | cytokine receptor activity | 103 | 0.001071 | 0.02008 |
| GO | GO:0007517 | muscle organ development | 453 | 0.001074 | 0.02008 |
| GO | GO:0003779 | actin binding | 404 | 0.001113 | 0.020572 |
| GO | GO:0055001 | muscle cell development | 185 | 0.001123 | 0.020572 |
| GO | GO:0070851 | growth factor receptor binding | 135 | 0.001162 | 0.021069 |
| GO | GO:0050953 | sensory perception of light stimulus | 227 | 0.001182 | 0.021224 |
| GO | GO:0030017 | sarcomere | 197 | 0.001276 | 0.022523 |
| GO | GO:0050866 | negative regulation of cell activation | 211 | 0.001286 | 0.022523 |
| GO | GO:0006024 | glycosaminoglycan biosynthetic process | 136 | 0.001292 | 0.022523 |
| GO | GO:0050863 | regulation of T cell activation | 373 | 0.001356 | 0.023406 |
| GO | GO:0048634 | regulation of muscle organ development | 172 | 0.001386 | 0.023699 |
| GO | GO:0007601 | visual perception | 219 | 0.001414 | 0.023953 |
| GO | GO:0016202 | regulation of striated muscle tissue development | 167 | 0.001434 | 0.024031 |
| GO | GO:0050678 | regulation of epithelial cell proliferation | 412 | 0.001445 | 0.024031 |
| GO | GO:0007204 | positive regulation of cytosolic calcium ion concentration | 318 | 0.001459 | 0.024053 |
| GO | GO:0043292 | contractile fiber | 252 | 0.001477 | 0.024119 |
| GO | GO:0032943 | mononuclear cell proliferation | 277 | 0.001503 | 0.024324 |
| GO | GO:0060419 | heart growth | 118 | 0.001568 | 0.025159 |
| GO | GO:0062013 | positive regulation of small molecule metabolic process | 162 | 0.00159 | 0.025231 |
| GO | GO:0006023 | aminoglycan biosynthetic process | 141 | 0.0016 | 0.025231 |
| GO | GO:0061138 | morphogenesis of a branching epithelium | 193 | 0.001648 | 0.025753 |
| GO | GO:0032612 | interleukin-1 production | 121 | 0.001671 | 0.02589 |
| GO | GO:0061448 | connective tissue development | 267 | 0.001695 | 0.025974 |
| GO | GO:0005788 | endoplasmic reticulum lumen | 316 | 0.001705 | 0.025974 |
| GO | GO:0017048 | Rho GTPase binding | 155 | 0.001757 | 0.026551 |
| GO | GO:0001935 | endothelial cell proliferation | 210 | 0.001779 | 0.026654 |
| GO | GO:0071222 | cellular response to lipopolysaccharide | 191 | 0.001794 | 0.02666 |
| GO | GO:0098742 | cell-cell adhesion via plasma-membrane adhesion molecules | 266 | 0.001875 | 0.027634 |
| GO | GO:0001523 | retinoid metabolic process | 129 | 0.001925 | 0.028149 |
| GO | GO:0005125 | cytokine activity | 175 | 0.001951 | 0.028304 |
| GO | GO:0010634 | positive regulation of epithelial cell migration | 200 | 0.002018 | 0.029034 |
| GO | GO:0050870 | positive regulation of T cell activation | 231 | 0.002059 | 0.029394 |
| GO | GO:0045444 | fat cell differentiation | 230 | 0.002083 | 0.02951 |
| GO | GO:0055002 | striated muscle cell development | 166 | 0.002238 | 0.031454 |
| GO | GO:0002040 | sprouting angiogenesis | 208 | 0.002292 | 0.031717 |
| GO | GO:0001936 | regulation of endothelial cell proliferation | 193 | 0.002296 | 0.031717 |
| GO | GO:1901861 | regulation of muscle tissue development | 171 | 0.002309 | 0.031717 |
| GO | GO:0001763 | morphogenesis of a branching structure | 208 | 0.002439 | 0.033127 |
| GO | GO:0030016 | myofibril | 234 | 0.002448 | 0.033127 |
| GO | GO:0046651 | lymphocyte proliferation | 273 | 0.002474 | 0.033223 |
| GO | GO:2000181 | negative regulation of blood vessel morphogenesis | 202 | 0.002506 | 0.033404 |
| GO | GO:0022408 | negative regulation of cell-cell adhesion | 188 | 0.002539 | 0.033602 |
| GO | GO:0008083 | growth factor activity | 150 | 0.002619 | 0.034294 |
| GO | GO:2000379 | positive regulation of reactive oxygen species metabolic process | 108 | 0.002639 | 0.034294 |
| GO | GO:1903038 | negative regulation of leukocyte cell-cell adhesion | 135 | 0.002648 | 0.034294 |
| GO | GO:0043491 | protein kinase B signaling | 293 | 0.00271 | 0.034846 |
| GO | GO:0002695 | negative regulation of leukocyte activation | 186 | 0.002744 | 0.035032 |
| GO | GO:0043583 | ear development | 218 | 0.002827 | 0.035847 |
| GO | GO:0016101 | diterpenoid metabolic process | 135 | 0.00287 | 0.036136 |
| GO | GO:0050731 | positive regulation of peptidyl-tyrosine phosphorylation | 204 | 0.002943 | 0.036798 |
| GO | GO:0010595 | positive regulation of endothelial cell migration | 149 | 0.003058 | 0.037413 |
| GO | GO:0042129 | regulation of T cell proliferation | 165 | 0.003073 | 0.037413 |
| GO | GO:0051896 | regulation of protein kinase B signaling | 258 | 0.003083 | 0.037413 |
| GO | GO:0051250 | negative regulation of lymphocyte activation | 149 | 0.003098 | 0.037413 |
| GO | GO:0070372 | regulation of ERK1 and ERK2 cascade | 294 | 0.003109 | 0.037413 |
| GO | GO:0050867 | positive regulation of cell activation | 374 | 0.003116 | 0.037413 |
| GO | GO:0004497 | monooxygenase activity | 138 | 0.003222 | 0.038428 |
| GO | GO:0002696 | positive regulation of leukocyte activation | 357 | 0.00328 | 0.038868 |
| GO | GO:0005516 | calmodulin binding | 179 | 0.003349 | 0.039431 |
| GO | GO:1903531 | negative regulation of secretion by cell | 217 | 0.003401 | 0.039782 |
| GO | GO:0019199 | transmembrane receptor protein kinase activity | 101 | 0.003447 | 0.040057 |
| GO | GO:0002237 | response to molecule of bacterial origin | 344 | 0.003522 | 0.040517 |
| GO | GO:0051048 | negative regulation of secretion | 241 | 0.003531 | 0.040517 |
| GO | GO:0048771 | tissue remodeling | 180 | 0.003614 | 0.041206 |
| GO | GO:0045667 | regulation of osteoblast differentiation | 130 | 0.003659 | 0.041466 |
| GO | GO:0007599 | hemostasis | 413 | 0.003895 | 0.043857 |
| GO | GO:0032649 | regulation of interferon-gamma production | 104 | 0.003978 | 0.044525 |
| GO | GO:0002573 | myeloid leukocyte differentiation | 229 | 0.004492 | 0.049964 |
| GO | GO:0007596 | blood coagulation | 406 | 0.004634 | 0.051229 |
| GO | GO:0043588 | skin development | 458 | 0.004771 | 0.052421 |
| GO | GO:0070665 | positive regulation of leukocyte proliferation | 146 | 0.005079 | 0.055474 |
| GO | GO:0050817 | coagulation | 414 | 0.005242 | 0.056905 |
| GO | GO:0071706 | tumor necrosis factor superfamily cytokine production | 183 | 0.005528 | 0.059655 |
| GO | GO:0043235 | receptor complex | 331 | 0.005578 | 0.059843 |
| GO | GO:0048738 | cardiac muscle tissue development | 250 | 0.00567 | 0.060294 |
| GO | GO:0051249 | regulation of lymphocyte activation | 491 | 0.005687 | 0.060294 |
| GO | GO:0051928 | positive regulation of calcium ion transport | 128 | 0.00585 | 0.061661 |
| GO | GO:0045177 | apical part of cell | 381 | 0.005976 | 0.062624 |
| GO | GO:0046718 | viral entry into host cell | 126 | 0.00609 | 0.063369 |
| GO | GO:0071216 | cellular response to biotic stimulus | 232 | 0.006124 | 0.063369 |
| GO | GO:1901681 | sulfur compound binding | 229 | 0.006152 | 0.063369 |
| GO | GO:0001704 | formation of primary germ layer | 126 | 0.006265 | 0.06417 |
| GO | GO:0072376 | protein activation cascade | 208 | 0.006358 | 0.064755 |
| GO | GO:0050868 | negative regulation of T cell activation | 116 | 0.00658 | 0.066612 |
| GO | GO:0016525 | negative regulation of angiogenesis | 199 | 0.006613 | 0.066612 |
| GO | GO:0051224 | negative regulation of protein transport | 194 | 0.006705 | 0.066955 |
| GO | GO:0034754 | cellular hormone metabolic process | 148 | 0.006721 | 0.066955 |
| GO | GO:0032963 | collagen metabolic process | 113 | 0.006808 | 0.067445 |
| GO | GO:1903034 | regulation of response to wounding | 195 | 0.006926 | 0.068248 |
| GO | GO:1903555 | regulation of tumor necrosis factor superfamily cytokine production | 176 | 0.007023 | 0.068825 |
| GO | GO:0030018 | Z disc | 110 | 0.007105 | 0.069251 |
| GO | GO:1903510 | mucopolysaccharide metabolic process | 142 | 0.007273 | 0.070513 |
| GO | GO:0030324 | lung development | 180 | 0.007539 | 0.072701 |
| GO | GO:0033002 | muscle cell proliferation | 278 | 0.007842 | 0.074515 |
| GO | GO:0030414 | peptidase inhibitor activity | 184 | 0.007843 | 0.074515 |
| GO | GO:0006721 | terpenoid metabolic process | 146 | 0.007887 | 0.074515 |
| GO | GO:0090257 | regulation of muscle system process | 304 | 0.007891 | 0.074515 |
| GO | GO:0031099 | regeneration | 197 | 0.008221 | 0.076975 |
| GO | GO:0032946 | positive regulation of mononuclear cell proliferation | 138 | 0.008237 | 0.076975 |
| GO | GO:0001655 | urogenital system development | 390 | 0.008371 | 0.077828 |
| GO | GO:0010876 | lipid localization | 472 | 0.00864 | 0.079804 |
| GO | GO:0042098 | T cell proliferation | 196 | 0.008671 | 0.079804 |
| GO | GO:0071559 | response to transforming growth factor beta | 300 | 0.009073 | 0.082729 |
| GO | GO:0019722 | calcium-mediated signaling | 230 | 0.009081 | 0.082729 |
| GO | GO:0007605 | sensory perception of sound | 142 | 0.009158 | 0.083015 |
| GO | GO:0032496 | response to lipopolysaccharide | 316 | 0.009402 | 0.084803 |
| GO | GO:0050730 | regulation of peptidyl-tyrosine phosphorylation | 278 | 0.009518 | 0.08543 |
| GO | GO:0005938 | cell cortex | 242 | 0.009613 | 0.085856 |
| GO | GO:0032640 | tumor necrosis factor production | 176 | 0.009905 | 0.087958 |
| GO | GO:0070374 | positive regulation of ERK1 and ERK2 cascade | 202 | 0.009946 | 0.087958 |
| GO | GO:0031674 | I band | 123 | 0.010056 | 0.0885 |
| GO | GO:0006936 | muscle contraction | 440 | 0.010242 | 0.088839 |
| GO | GO:0030260 | entry into host cell | 142 | 0.010342 | 0.088839 |
| GO | GO:0044409 | entry into host | 142 | 0.010342 | 0.088839 |
| GO | GO:0051806 | entry into cell of other organism involved in symbiotic interaction | 142 | 0.010342 | 0.088839 |
| GO | GO:0051828 | entry into other organism involved in symbiotic interaction | 142 | 0.010342 | 0.088839 |
| GO | GO:0070371 | ERK1 and ERK2 cascade | 319 | 0.010388 | 0.088839 |
| GO | GO:0032970 | regulation of actin filament-based process | 404 | 0.010593 | 0.090163 |
| GO | GO:0017015 | regulation of transforming growth factor beta receptor signaling pathway | 135 | 0.010937 | 0.092475 |
| GO | GO:0038024 | cargo receptor activity | 107 | 0.010992 | 0.092475 |
| GO | GO:0007015 | actin filament organization | 423 | 0.011024 | 0.092475 |
| GO | GO:0007266 | Rho protein signal transduction | 171 | 0.011068 | 0.092475 |
| GO | GO:0042692 | muscle cell differentiation | 433 | 0.01124 | 0.093457 |
| GO | GO:0051015 | actin filament binding | 153 | 0.011289 | 0.093457 |
| GO | GO:0030175 | filopodium | 103 | 0.011341 | 0.093457 |
| GO | GO:1904950 | negative regulation of establishment of protein localization | 198 | 0.011509 | 0.094251 |
| GO | GO:0004252 | serine-type endopeptidase activity | 154 | 0.011548 | 0.094251 |
| GO | GO:0050714 | positive regulation of protein secretion | 286 | 0.011593 | 0.094251 |
| GO | GO:0001938 | positive regulation of endothelial cell proliferation | 117 | 0.011658 | 0.094357 |
| GO | GO:0035023 | regulation of Rho protein signal transduction | 129 | 0.012408 | 0.099979 |
| GO | GO:0045834 | positive regulation of lipid metabolic process | 155 | 0.012917 | 0.103298 |
| GO | GO:1903670 | regulation of sprouting angiogenesis | 165 | 0.012934 | 0.103298 |
| GO | GO:0043122 | regulation of I-kappaB kinase/NF-kappaB signaling | 247 | 0.013019 | 0.103523 |
| GO | GO:0042471 | ear morphogenesis | 118 | 0.013391 | 0.106016 |
| GO | GO:0042107 | cytokine metabolic process | 136 | 0.013663 | 0.107702 |
| GO | GO:0032609 | interferon-gamma production | 121 | 0.014176 | 0.111258 |
| GO | GO:0071560 | cellular response to transforming growth factor beta stimulus | 292 | 0.01432 | 0.111909 |
| GO | GO:0032680 | regulation of tumor necrosis factor production | 171 | 0.014953 | 0.116351 |
| GO | GO:0022604 | regulation of cell morphogenesis | 462 | 0.015084 | 0.116869 |
| GO | GO:0030323 | respiratory tube development | 184 | 0.015452 | 0.119164 |
| GO | GO:0030048 | actin filament-based movement | 157 | 0.015512 | 0.119164 |
| GO | GO:0034330 | cell junction organization | 323 | 0.01601 | 0.122475 |
| GO | GO:0050671 | positive regulation of lymphocyte proliferation | 135 | 0.016221 | 0.123568 |
| GO | GO:0022612 | gland morphogenesis | 118 | 0.016302 | 0.123666 |
| GO | GO:0006805 | xenobiotic metabolic process | 118 | 0.016546 | 0.124994 |
| GO | GO:0055007 | cardiac muscle cell differentiation | 134 | 0.016706 | 0.125673 |
| GO | GO:0043648 | dicarboxylic acid metabolic process | 113 | 0.017094 | 0.12695 |
| GO | GO:0061041 | regulation of wound healing | 163 | 0.017131 | 0.12695 |
| GO | GO:0015849 | organic acid transport | 381 | 0.017219 | 0.12695 |
| GO | GO:0046942 | carboxylic acid transport | 381 | 0.017219 | 0.12695 |
| GO | GO:1903844 | regulation of cellular response to transforming growth factor beta stimulus | 139 | 0.017225 | 0.12695 |
| GO | GO:0043535 | regulation of blood vessel endothelial cell migration | 178 | 0.017672 | 0.129365 |
| GO | GO:1902105 | regulation of leukocyte differentiation | 308 | 0.017696 | 0.129365 |
| GO | GO:0016323 | basolateral plasma membrane | 205 | 0.017862 | 0.130057 |
| GO | GO:1903169 | regulation of calcium ion transmembrane transport | 185 | 0.018083 | 0.131141 |
| GO | GO:0046620 | regulation of organ growth | 117 | 0.019103 | 0.13798 |
| GO | GO:0001823 | mesonephros development | 117 | 0.019291 | 0.138352 |
| GO | GO:0070252 | actin-mediated cell contraction | 135 | 0.019307 | 0.138352 |
| GO | GO:0010596 | negative regulation of endothelial cell migration | 111 | 0.019994 | 0.14271 |
| GO | GO:0032611 | interleukin-1 beta production | 102 | 0.020139 | 0.143187 |
| GO | GO:0042446 | hormone biosynthetic process | 102 | 0.020576 | 0.14572 |
| GO | GO:0043123 | positive regulation of I-kappaB kinase/NF-kappaB signaling | 189 | 0.020731 | 0.146245 |
| GO | GO:0006022 | aminoglycan metabolic process | 214 | 0.020866 | 0.146627 |
| GO | GO:0002793 | positive regulation of peptide secretion | 312 | 0.021107 | 0.147453 |
| GO | GO:0030098 | lymphocyte differentiation | 375 | 0.021146 | 0.147453 |
| GO | GO:0050729 | positive regulation of inflammatory response | 170 | 0.021464 | 0.148613 |
| GO | GO:0055024 | regulation of cardiac muscle tissue development | 112 | 0.021476 | 0.148613 |
| GO | GO:0030203 | glycosaminoglycan metabolic process | 204 | 0.021808 | 0.150335 |
| GO | GO:0032956 | regulation of actin cytoskeleton organization | 353 | 0.022293 | 0.152678 |
| GO | GO:0030667 | secretory granule membrane | 309 | 0.022338 | 0.152678 |
| GO | GO:0060048 | cardiac muscle contraction | 161 | 0.022405 | 0.152678 |
| GO | GO:1902903 | regulation of supramolecular fiber organization | 367 | 0.022534 | 0.152678 |
| GO | GO:0007249 | I-kappaB kinase/NF-kappaB signaling | 304 | 0.022569 | 0.152678 |
| GO | GO:0035051 | cardiocyte differentiation | 182 | 0.02321 | 0.15643 |
| GO | GO:0048638 | regulation of developmental growth | 343 | 0.024132 | 0.161839 |
| GO | GO:0051251 | positive regulation of lymphocyte activation | 304 | 0.024191 | 0.161839 |
| GO | GO:0016324 | apical plasma membrane | 310 | 0.02438 | 0.162046 |
| GO | GO:0014812 | muscle cell migration | 110 | 0.024431 | 0.162046 |
| GO | GO:0032652 | regulation of interleukin-1 production | 106 | 0.02449 | 0.162046 |
| GO | GO:0051216 | cartilage development | 201 | 0.024976 | 0.164209 |
| GO | GO:0043534 | blood vessel endothelial cell migration | 206 | 0.025036 | 0.164209 |
| GO | GO:0004866 | endopeptidase inhibitor activity | 173 | 0.025089 | 0.164209 |
| GO | GO:1903035 | negative regulation of response to wounding | 100 | 0.025636 | 0.16719 |
| GO | GO:0010466 | negative regulation of peptidase activity | 235 | 0.025844 | 0.167939 |
| GO | GO:0072001 | renal system development | 351 | 0.026342 | 0.170562 |
| GO | GO:0042089 | cytokine biosynthetic process | 135 | 0.026464 | 0.170747 |
| GO | GO:0034308 | primary alcohol metabolic process | 100 | 0.026745 | 0.171947 |
| GO | GO:0030168 | platelet activation | 174 | 0.028098 | 0.180005 |
| GO | GO:0005342 | organic acid transmembrane transporter activity | 141 | 0.028688 | 0.182273 |
| GO | GO:0004175 | endopeptidase activity | 412 | 0.028742 | 0.182273 |
| GO | GO:0031032 | actomyosin structure organization | 182 | 0.028897 | 0.182273 |
| GO | GO:0046632 | alpha-beta T cell differentiation | 109 | 0.028996 | 0.182273 |
| GO | GO:0048010 | vascular endothelial growth factor receptor signaling pathway | 111 | 0.029016 | 0.182273 |
| GO | GO:0016298 | lipase activity | 136 | 0.029124 | 0.182273 |
| GO | GO:0046328 | regulation of JNK cascade | 195 | 0.029156 | 0.182273 |
| GO | GO:0042035 | regulation of cytokine biosynthetic process | 123 | 0.030337 | 0.189007 |
| GO | GO:0007369 | gastrulation | 196 | 0.03098 | 0.191803 |
| GO | GO:0006869 | lipid transport | 422 | 0.030997 | 0.191803 |
| GO | GO:0002020 | protease binding | 123 | 0.03123 | 0.191922 |
| GO | GO:0061135 | endopeptidase regulator activity | 178 | 0.031373 | 0.191922 |
| GO | GO:1902904 | negative regulation of supramolecular fiber organization | 132 | 0.031439 | 0.191922 |
| GO | GO:0009612 | response to mechanical stimulus | 208 | 0.03144 | 0.191922 |
| GO | GO:0002761 | regulation of myeloid leukocyte differentiation | 122 | 0.031613 | 0.192329 |
| GO | GO:0007229 | integrin-mediated signaling pathway | 101 | 0.031948 | 0.193458 |
| GO | GO:0046631 | alpha-beta T cell activation | 153 | 0.032113 | 0.193458 |
| GO | GO:0003158 | endothelium development | 145 | 0.032118 | 0.193458 |
| GO | GO:0006814 | sodium ion transport | 231 | 0.03247 | 0.194655 |
| GO | GO:0051146 | striated muscle cell differentiation | 306 | 0.032532 | 0.194655 |
| GO | GO:0045598 | regulation of fat cell differentiation | 132 | 0.033109 | 0.196775 |
| GO | GO:0061326 | renal tubule development | 108 | 0.0332 | 0.196775 |
| GO | GO:0046943 | carboxylic acid transmembrane transporter activity | 140 | 0.033212 | 0.196775 |
| GO | GO:0030217 | T cell differentiation | 275 | 0.033479 | 0.197258 |
| GO | GO:0003014 | renal system process | 135 | 0.033511 | 0.197258 |
| GO | GO:0019897 | extrinsic component of plasma membrane | 104 | 0.034459 | 0.20213 |
| GO | GO:0001894 | tissue homeostasis | 223 | 0.034562 | 0.20213 |
| GO | GO:0098552 | side of membrane | 366 | 0.034674 | 0.202137 |
| GO | GO:0043271 | negative regulation of ion transport | 155 | 0.035473 | 0.206131 |
| GO | GO:0048762 | mesenchymal cell differentiation | 247 | 0.036107 | 0.2086 |
| GO | GO:0004222 | metalloendopeptidase activity | 106 | 0.036128 | 0.2086 |
| GO | GO:1903532 | positive regulation of secretion by cell | 433 | 0.036985 | 0.212867 |
| GO | GO:0006941 | striated muscle contraction | 211 | 0.037286 | 0.213922 |
| GO | GO:0016485 | protein processing | 331 | 0.037588 | 0.214757 |
| GO | GO:0034766 | negative regulation of ion transmembrane transport | 104 | 0.037668 | 0.214757 |
| GO | GO:0051147 | regulation of muscle cell differentiation | 212 | 0.038695 | 0.219919 |
| GO | GO:1903426 | regulation of reactive oxygen species biosynthetic process | 106 | 0.039082 | 0.221423 |
| GO | GO:0042136 | neurotransmitter biosynthetic process | 106 | 0.039776 | 0.224651 |
| GO | GO:0001822 | kidney development | 329 | 0.04027 | 0.226516 |
| GO | GO:0090288 | negative regulation of cellular response to growth factor stimulus | 183 | 0.040356 | 0.226516 |
| GO | GO:0044706 | multi-multicellular organism process | 232 | 0.040559 | 0.226954 |
| GO | GO:0005088 | Ras guanyl-nucleotide exchange factor activity | 145 | 0.040976 | 0.228584 |
| GO | GO:0061134 | peptidase regulator activity | 220 | 0.042009 | 0.233628 |
| GO | GO:0048732 | gland development | 453 | 0.042703 | 0.236019 |
| GO | GO:0030216 | keratinocyte differentiation | 322 | 0.042833 | 0.236019 |
| GO | GO:0060538 | skeletal muscle organ development | 177 | 0.042837 | 0.236019 |
| GO | GO:0032675 | regulation of interleukin-6 production | 152 | 0.04296 | 0.236019 |
| GO | GO:0050715 | positive regulation of cytokine secretion | 149 | 0.043236 | 0.236821 |
| GO | GO:0070613 | regulation of protein processing | 188 | 0.043539 | 0.237757 |
| GO | GO:0051017 | actin filament bundle assembly | 153 | 0.044309 | 0.240514 |
| GO | GO:0061572 | actin filament bundle organization | 154 | 0.044309 | 0.240514 |
| GO | GO:0002062 | chondrocyte differentiation | 101 | 0.044605 | 0.241399 |
| GO | GO:0090092 | regulation of transmembrane receptor protein serine/threonine kinase signaling pathway | 269 | 0.045795 | 0.247103 |
| GO | GO:0032635 | interleukin-6 production | 163 | 0.047974 | 0.25809 |
| GO | GO:0050954 | sensory perception of mechanical stimulus | 162 | 0.04878 | 0.261651 |
| GO | GO:0048754 | branching morphogenesis of an epithelial tube | 157 | 0.049354 | 0.26395 |
| GO | GO:0007565 | female pregnancy | 198 | 0.049665 | 0.264626 |
| GO | GO:0002819 | regulation of adaptive immune response | 176 | 0.049781 | 0.264626 |
| GO | GO:0007179 | transforming growth factor beta receptor signaling pathway | 236 | 0.049918 | 0.264626 |
