# Supplementary material for: TWIST1 DNA methylation is a cell marker of airway and parenchymal lung fibroblasts that are differentially methylated in asthma
Source: Clin Epigenetics. 2020 Oct 2;12:145. doi: 10.1186/s13148-020-00931-4 (PMC7531162; doi:10.1186/s13148-020-00931-4)
Supplement: Supplementary file 2 — Additional file 2. Supplementary Tables 1-8 [file 13148_2020_931_MOESM2_ESM.zip › ST3.docx]

**Supplemental Table 3: Summary of 42 genes that display differential gene expression associated with differential methylation of at least one DNA methylation probe**

| Gene | Corresponding Number of CpGs | CpG Site | Delta Beta | CpG Bonferroni FWER | log2 Fold Change | Gene Expression Bonferroni FWER |
| --- | --- | --- | --- | --- | --- | --- |
| TBX18 | 1 | cg09817427 | 0.152 | 2.89E-05 | -3.475 | 2.04E-10 |
| EYA1 | 5 | cg16808120 | -0.475 | 0.002104173 | -3.223 | 2.12E-09 |
| EYA1 | 5 | cg08234354 | -0.391 | 8.49E-05 | -3.223 | 2.12E-09 |
| EYA1 | 5 | cg21312929 | -0.339 | 4.79E-05 | -3.223 | 2.12E-09 |
| EYA1 | 5 | cg03090171 | -0.283 | 0.005620137 | -3.223 | 2.12E-09 |
| EYA1 | 5 | cg06693048 | -0.174 | 0.008928554 | -3.223 | 2.12E-09 |
| FNDC1 | 2 | cg14500486 | -0.512 | 0.048577336 | -2.867 | 0.000748877 |
| FNDC1 | 2 | cg00219282 | -0.272 | 0.004535254 | -2.867 | 0.000748877 |
| OSR2 | 16 | cg07358855 | 0.107 | 0.019350509 | -2.753 | 3.85E-07 |
| OSR2 | 16 | cg22920700 | 0.230 | 0.000195018 | -2.753 | 3.85E-07 |
| OSR2 | 16 | cg04768602 | 0.230 | 3.87E-05 | -2.753 | 3.85E-07 |
| OSR2 | 16 | cg00388812 | 0.243 | 0.040401115 | -2.753 | 3.85E-07 |
| OSR2 | 16 | cg13016314 | 0.280 | 0.001888159 | -2.753 | 3.85E-07 |
| OSR2 | 16 | cg08115114 | 0.297 | 2.19E-05 | -2.753 | 3.85E-07 |
| OSR2 | 16 | cg01148781 | 0.307 | 0.034242502 | -2.753 | 3.85E-07 |
| OSR2 | 16 | cg18744668 | 0.313 | 0.001980473 | -2.753 | 3.85E-07 |
| OSR2 | 16 | cg16746576 | 0.314 | 0.006058197 | -2.753 | 3.85E-07 |
| OSR2 | 16 | cg05754861 | 0.344 | 0.001066511 | -2.753 | 3.85E-07 |
| OSR2 | 16 | cg14548272 | 0.351 | 0.005726307 | -2.753 | 3.85E-07 |
| OSR2 | 16 | cg08202494 | 0.359 | 0.001436057 | -2.753 | 3.85E-07 |
| OSR2 | 16 | cg03157027 | 0.413 | 3.35E-07 | -2.753 | 3.85E-07 |
| OSR2 | 16 | cg14657725 | 0.503 | 1.93E-06 | -2.753 | 3.85E-07 |
| OSR2 | 16 | cg23820828 | 0.518 | 2.34E-07 | -2.753 | 3.85E-07 |
| OSR2 | 16 | cg22524061 | 0.663 | 3.59E-08 | -2.753 | 3.85E-07 |
| TWIST2 | 7 | cg02281489 | 0.195 | 0.003728078 | -2.593 | 0.00058314 |
| TWIST2 | 7 | cg04840356 | 0.308 | 0.000406542 | -2.593 | 0.00058314 |
| TWIST2 | 7 | cg15548656 | 0.308 | 1.18E-05 | -2.593 | 0.00058314 |
| TWIST2 | 7 | cg17253741 | 0.320 | 0.001720874 | -2.593 | 0.00058314 |
| TWIST2 | 7 | cg19965312 | 0.394 | 0.001106308 | -2.593 | 0.00058314 |
| TWIST2 | 7 | cg06738242 | 0.414 | 0.000246917 | -2.593 | 0.00058314 |
| TWIST2 | 7 | cg26913248 | 0.436 | 0.007477499 | -2.593 | 0.00058314 |
| CRLF1 | 2 | cg24125901 | -0.526 | 5.45E-06 | -2.299 | 0.006074528 |
| CRLF1 | 2 | cg04072597 | -0.463 | 0.007556495 | -2.299 | 0.006074528 |
| FBLN2 | 3 | cg08014106 | -0.132 | 0.036299444 | -2.168 | 5.80E-05 |
| FBLN2 | 3 | cg26021633 | -0.120 | 0.04857478 | -2.168 | 5.80E-05 |
| FBLN2 | 3 | cg25120326 | 0.390 | 0.020888839 | -2.168 | 5.80E-05 |
| ABCA9 | 1 | cg06213598 | 0.380 | 0.000171606 | -2.160 | 2.64E-07 |
| ADAM33 | 1 | cg23073319 | 0.489 | 9.96E-05 | -2.107 | 2.18E-05 |
| TSPAN11 | 1 | cg15526671 | 0.343 | 0.002592784 | -1.925 | 0.005277734 |
| NTN1 | 4 | cg07114544 | -0.364 | 0.002785923 | -1.787 | 0.000194366 |
| NTN1 | 4 | cg02481697 | 0.293 | 0.044249704 | -1.787 | 0.000194366 |
| NTN1 | 4 | cg09935792 | 0.316 | 0.020562737 | -1.787 | 0.000194366 |
| NTN1 | 4 | cg01862363 | 0.380 | 0.040234183 | -1.787 | 0.000194366 |
| MN1 | 4 | cg20680669 | 0.210 | 0.007096032 | -1.739 | 0.013241026 |
| MN1 | 4 | cg22976732 | 0.395 | 1.05E-05 | -1.739 | 0.013241026 |
| MN1 | 4 | cg06004017 | 0.477 | 1.57E-06 | -1.739 | 0.013241026 |
| MN1 | 4 | cg00187327 | 0.488 | 6.06E-09 | -1.739 | 0.013241026 |
| AKAP12 | 1 | cg24121503 | 0.299 | 0.026460963 | -1.717 | 0.000765126 |
| SH3BP5 | 3 | cg18444702 | 0.335 | 0.000113361 | -1.572 | 4.39E-08 |
| SH3BP5 | 3 | cg13279476 | 0.355 | 0.003739999 | -1.572 | 4.39E-08 |
| SH3BP5 | 3 | cg02275418 | 0.434 | 0.000192801 | -1.572 | 4.39E-08 |
| ARHGAP26 | 1 | cg22184990 | -0.490 | 0.000193288 | -1.393 | 0.000196237 |
| FBN1 | 3 | cg14549078 | 0.254 | 0.020643252 | -1.391 | 0.034837609 |
| FBN1 | 3 | cg10095534 | 0.337 | 0.040334796 | -1.391 | 0.034837609 |
| FBN1 | 3 | cg24631427 | 0.429 | 0.019072233 | -1.391 | 0.034837609 |
| SYNE2 | 1 | cg10018167 | -0.115 | 0.022059532 | -1.288 | 0.02883392 |
| EPHB2 | 1 | cg12717584 | 0.226 | 0.00095277 | -1.150 | 0.022862568 |
| FBLN1 | 1 | cg25243775 | 0.286 | 0.025427826 | -1.139 | 0.015712391 |
| IRS2 | 1 | cg01569664 | 0.537 | 0.001239688 | -1.137 | 0.037492701 |
| ZFHX4 | 3 | cg00937330 | -0.386 | 1.79E-06 | -1.122 | 0.002611649 |
| ZFHX4 | 3 | cg15168043 | -0.365 | 4.62E-07 | -1.122 | 0.002611649 |
| ZFHX4 | 3 | cg16877004 | -0.266 | 0.001750518 | -1.122 | 0.002611649 |
| NFATC4 | 3 | cg01580176 | 0.262 | 0.02601241 | -1.084 | 0.001787199 |
| NFATC4 | 3 | cg05243890 | 0.279 | 0.032330815 | -1.084 | 0.001787199 |
| NFATC4 | 3 | cg16371477 | 0.473 | 0.00010389 | -1.084 | 0.001787199 |
| PFKFB3 | 1 | cg18262201 | -0.192 | 0.000724068 | -1.044 | 0.026392444 |
| NFIA | 1 | cg26252077 | -0.361 | 0.001319047 | -1.027 | 0.000273219 |
| LRRK1 | 1 | cg16701059 | -0.364 | 0.000346675 | -0.995 | 0.009676774 |
| PLEKHA5 | 1 | cg21140028 | 0.313 | 7.46E-05 | -0.854 | 0.002022355 |
| IGF1R | 1 | cg25404375 | -0.159 | 0.03674869 | -0.846 | 0.006203544 |
| VPS13B | 1 | cg17117823 | 0.419 | 1.89E-05 | -0.826 | 0.011051985 |
| PRKCE | 1 | cg22549086 | -0.379 | 3.42E-07 | -0.824 | 0.00556497 |
| EPHX1 | 1 | cg25152404 | -0.277 | 0.004517154 | -0.819 | 0.003537407 |
| CDC25B | 1 | cg02737268 | 0.366 | 0.002530183 | -0.785 | 0.044900448 |
| SNX29 | 1 | cg00548009 | 0.141 | 0.002290785 | -0.772 | 0.048129847 |
| SKAP2 | 5 | cg03730533 | -0.657 | 3.45E-07 | 0.862 | 0.001035895 |
| SKAP2 | 5 | cg12140851 | -0.582 | 8.37E-06 | 0.862 | 0.001035895 |
| SKAP2 | 5 | cg12252069 | -0.544 | 4.03E-05 | 0.862 | 0.001035895 |
| SKAP2 | 5 | cg10180281 | -0.514 | 0.000103083 | 0.862 | 0.001035895 |
| SKAP2 | 5 | cg20747577 | -0.390 | 0.037447387 | 0.862 | 0.001035895 |
| MFSD6 | 1 | cg16924045 | -0.307 | 0.024167324 | 1.046 | 0.003066156 |
| DDO | 5 | cg02872426 | -0.497 | 0.00131232 | 1.170 | 0.000562383 |
| DDO | 5 | cg06413398 | -0.253 | 8.56E-05 | 1.170 | 0.000562383 |
| DDO | 5 | cg07164639 | -0.210 | 0.004358472 | 1.170 | 0.000562383 |
| DDO | 5 | cg00804078 | -0.151 | 0.0236081 | 1.170 | 0.000562383 |
| DDO | 5 | cg12618475 | 0.131 | 0.007925155 | 1.170 | 0.000562383 |
| TSHZ2 | 2 | cg09566894 | 0.138 | 0.004347885 | 1.564 | 0.031778268 |
| TSHZ2 | 2 | cg20265403 | 0.212 | 0.022163648 | 1.564 | 0.031778268 |
| UBASH3B | 1 | cg25943661 | -0.444 | 9.91E-06 | 1.669 | 0.000856758 |
| JUP | 1 | cg14131889 | 0.113 | 0.001520735 | 1.844 | 0.000673052 |
| SRGN | 1 | cg02851793 | -0.229 | 0.005351891 | 2.087 | 0.008877165 |
| CD4 | 6 | cg13512987 | -0.414 | 1.85E-05 | 2.254 | 2.82E-06 |
| CD4 | 6 | cg22908581 | -0.322 | 1.83E-07 | 2.254 | 2.82E-06 |
| CD4 | 6 | cg05044173 | -0.304 | 0.00054037 | 2.254 | 2.82E-06 |
| CD4 | 6 | cg06624527 | -0.272 | 0.011865742 | 2.254 | 2.82E-06 |
| CD4 | 6 | cg24219128 | 0.078 | 0.001522054 | 2.254 | 2.82E-06 |
| CD4 | 6 | cg16875554 | 0.151 | 5.04E-07 | 2.254 | 2.82E-06 |
| CDH13 | 5 | cg05847519 | 0.127 | 0.001854011 | 3.094 | 0.00054933 |
| CDH13 | 5 | cg16387516 | 0.142 | 0.001682661 | 3.094 | 0.00054933 |
| CDH13 | 5 | cg26735980 | 0.188 | 0.016451433 | 3.094 | 0.00054933 |
| CDH13 | 5 | cg07092029 | 0.237 | 0.004639406 | 3.094 | 0.00054933 |
| CDH13 | 5 | cg01750200 | 0.260 | 0.008630562 | 3.094 | 0.00054933 |
| VAT1L | 2 | cg04378874 | 0.187 | 0.000878273 | 3.809 | 1.10E-05 |
| VAT1L | 2 | cg01169873 | 0.221 | 0.033097965 | 3.809 | 1.10E-05 |
