# Supplementary material for: TWIST1 DNA methylation is a cell marker of airway and parenchymal lung fibroblasts that are differentially methylated in asthma
Source: Clin Epigenetics. 2020 Oct 2;12:145. doi: 10.1186/s13148-020-00931-4 (PMC7531162; doi:10.1186/s13148-020-00931-4)
Supplement: Supplementary file 2 — Additional file 2. Supplementary Tables 1-8 [file 13148_2020_931_MOESM2_ESM.zip › ST4.docx]

**Supplementary Table 4: Summary of gene set enrichment testing for genes differentially methylated and expressed between airway and parenchymal fibroblasts.**

| Category | Term | Count | P Value | Genes |
| --- | --- | --- | --- | --- |
| GOTERM_MF_DIRECT | GO:0005201~extracellular matrix structural constituent | 4 | 3.88E-04 | FBLN1, FBLN2, FBN1, CD4 |
| GOTERM_CC_DIRECT | GO:0031012~extracellular matrix | 5 | 0.003 | JUP, TSHZ2, FBLN1, FBLN2, FBN1 |
| REACTOME_PATHWAY | R-HSA-2129379:R-HSA-2129379 | 3 | 0.003 | FBLN1, FBLN2, FBN1 |
| GOTERM_CC_DIRECT | GO:0005578~proteinaceous extracellular matrix | 3 | 0.107 | FBLN1, FBLN2, FBN1 |
| GOTERM_MF_DIRECT | GO:0005509~calcium ion binding | 4 | 0.195 | CDH13, FBLN1, FBLN2, FBN1 |
| GOTERM_CC_DIRECT | GO:0070062~extracellular exosome | 7 | 0.543 | JUP, CDH13, FBLN1, SNX29, SYNE2, FBLN2, FBN1 |
