# Supplementary material for: TWIST1 DNA methylation is a cell marker of airway and parenchymal lung fibroblasts that are differentially methylated in asthma
Source: Clin Epigenetics. 2020 Oct 2;12:145. doi: 10.1186/s13148-020-00931-4 (PMC7531162; doi:10.1186/s13148-020-00931-4)
Supplement: Supplementary file 2 — Additional file 2. Supplementary Tables 1-8 [file 13148_2020_931_MOESM2_ESM.zip › ST5.docx]

**Supplementary Table 5: Summary of the 17 regions identified by DMRcate as differentially methylated between airway fibroblasts isolated from individuals with and without asthma.**

| Chr | Start | End | Width | No. CpGs | Min.  FDR | Max.  Beta diff. | Mean Beta diff. | Gene |
| --- | --- | --- | --- | --- | --- | --- | --- | --- |
| chr1 | 53528612 | 53528991 | 380 | 3 | 4.41E-07 | 0.22 | 0.17 | PODN |
| chr1 | 234871410 | 234871477 | 68 | 3 | 1.67E-06 | 0.24 | 0.18 | NA |
| chr12 | 54446019 | 54446308 | 290 | 7 | 8.10E-07 | -0.20 | -0.14 | HOXC4 |
| chr12 | 121416315 | 121416796 | 482 | 9 | 9.27E-07 | 0.23 | 0.11 | HNF1A, HNF1A-AS1 |
| chr16 | 1080144 | 1080808 | 665 | 5 | 1.17E-08 | 0.15 | 0.06 | NA |
| chr16 | 86549237 | 86549962 | 726 | 5 | 3.94E-07 | 0.27 | 0.25 | NA |
| chr17 | 14206572 | 14207530 | 959 | 10 | 1.76E-07 | 0.21 | 0.14 | RP11-214O1.2 |
| chr19 | 12876947 | 12877188 | 242 | 3 | 5.06E-06 | -0.37 | -0.26 | HOOK2 |
| chr4 | 1166270 | 1167239 | 970 | 13 | 5.32E-08 | 0.15 | 0.11 | SPON2 |
| chr4 | 74847100 | 74848016 | 917 | 9 | 1.75E-08 | 0.17 | 0.12 | PF4 |
| chr5 | 2753852 | 2754240 | 389 | 7 | 1.07E-05 | -0.23 | -0.17 | C5orf38, IRX2 |
| chr5 | 172672454 | 172672684 | 231 | 2 | 1.41E-05 | 0.09 | 0.05 | NA |
| chr6 | 31695903 | 31696482 | 580 | 20 | 1.96E-06 | 0.11 | 0.06 | DDAH2 |
| chr6 | 33245328 | 33245895 | 568 | 25 | 9.27E-07 | 0.10 | 0.05 | B3GALT4, RPS18 |
| chr7 | 27163820 | 27163929 | 110 | 2 | 5.45E-06 | 0.14 | 0.11 | HOXA-AS2 |
| chr7 | 27197455 | 27198429 | 975 | 10 | 1.44E-07 | 0.22 | 0.13 | HOXA7, RP1-170O19.21 |
| chr8 | 599963 | 600233 | 271 | 3 | 2.19E-06 | -0.21 | -0.17 | NA |
