# Supplementary material for: TWIST1 DNA methylation is a cell marker of airway and parenchymal lung fibroblasts that are differentially methylated in asthma
Source: Clin Epigenetics. 2020 Oct 2;12:145. doi: 10.1186/s13148-020-00931-4 (PMC7531162; doi:10.1186/s13148-020-00931-4)
Supplement: Supplementary file 2 — Additional file 2. Supplementary Tables 1-8 [file 13148_2020_931_MOESM2_ESM.zip › ST6.docx]

**Supplementary Table 6: Summary of the 112 regions identified by DMRcate as differentially methylated between parenchymal fibroblasts isolated from individuals with and without asthma.**

| Chr | Start | End | Width | No. CpGs | Min.  FDR | Max.  Beta diff. | Mean Beta diff | Gene |
| --- | --- | --- | --- | --- | --- | --- | --- | --- |
| chr1 | 2004968 | 2006032 | 1065 | 9 | 3.04E-09 | 0.14 | 0.09 | PRKCZ |
| chr1 | 27961334 | 27962037 | 704 | 8 | 2.10E-09 | -0.17 | -0.09 | FGR |
| chr1 | 35220710 | 35220839 | 130 | 3 | 1.47E-06 | 0.14 | 0.09 | GJB5 |
| chr1 | 36574795 | 36574854 | 60 | 2 | 7.81E-08 | 0.15 | 0.14 | NA |
| chr1 | 47909234 | 47912079 | 2846 | 15 | 5.01E-26 | -0.20 | -0.12 | NA |
| chr1 | 50893149 | 50893469 | 321 | 3 | 6.56E-07 | -0.09 | -0.04 | NA |
| chr1 | 60539135 | 60539932 | 798 | 9 | 2.77E-15 | -0.12 | -0.05 | C1orf87 |
| chr1 | 86047911 | 86048923 | 1013 | 5 | 1.92E-14 | 0.17 | 0.09 | CYR61 |
| chr1 | 1.52E+08 | 1.52E+08 | 569 | 4 | 9.60E-14 | 0.23 | 0.16 | HRNR |
| chr1 | 1.59E+08 | 1.59E+08 | 455 | 3 | 3.77E-08 | 0.07 | 0.04 | DARC, CTA-134P22.2 |
| chr10 | 8095960 | 8097331 | 1372 | 28 | 8.72E-26 | -0.12 | -0.03 | GATA3, GATA3-AS1, RP11-379F12.3 |
| chr10 | 22633916 | 22634602 | 687 | 12 | 1.08E-08 | -0.08 | -0.03 | SPAG6 |
| chr10 | 35195138 | 35195758 | 621 | 4 | 4.07E-08 | -0.14 | -0.10 | NA |
| chr10 | 73848320 | 73849167 | 848 | 12 | 1.89E-09 | -0.13 | -0.07 | SPOCK2 |
| chr10 | 77163983 | 77165673 | 1691 | 10 | 3.23E-13 | -0.18 | -0.13 | ZNF503-AS2 |
| chr10 | 82295394 | 82295723 | 330 | 3 | 5.54E-07 | 0.13 | 0.10 | SH2D4B, RP11-137H2.4 |
| chr10 | 1.35E+08 | 1.35E+08 | 124 | 2 | 5.13E-07 | 0.10 | 0.09 | KNDC1 |
| chr11 | 1.16E+08 | 1.16E+08 | 33 | 2 | 2.38E-06 | -0.21 | -0.21 | AP000797.3 |
| chr11 | 1.18E+08 | 1.18E+08 | 68 | 2 | 2.22E-06 | 0.09 | 0.08 | AMICA1 |
| chr12 | 42876523 | 42876885 | 363 | 3 | 2.33E-10 | 0.01 | 0.00 | PRICKLE1 |
| chr12 | 54070891 | 54071194 | 304 | 7 | 4.82E-07 | -0.05 | -0.03 | ATP5G2 |
| chr12 | 1.16E+08 | 1.16E+08 | 310 | 3 | 6.49E-07 | -0.21 | -0.13 | NA |
| chr13 | 1.03E+08 | 1.03E+08 | 357 | 4 | 8.30E-08 | -0.19 | -0.14 | FGF14 |
| chr13 | 1.14E+08 | 1.14E+08 | 241 | 4 | 2.43E-07 | 0.16 | 0.10 | PCID2 |
| chr13 | 1.14E+08 | 1.14E+08 | 142 | 3 | 1.79E-06 | -0.13 | -0.12 | LAMP1 |
| chr14 | 38724648 | 38725312 | 665 | 4 | 4.67E-10 | -0.16 | -0.11 | CLEC14A |
| chr14 | 73361747 | 73362214 | 468 | 4 | 1.08E-07 | -0.15 | -0.06 | DPF3 |
| chr14 | 1E+08 | 1E+08 | 306 | 4 | 8.27E-07 | -0.17 | -0.11 | CCDC85C, RP11-543C4.1 |
| chr15 | 37180325 | 37180889 | 565 | 5 | 1.25E-07 | -0.15 | -0.09 | RP11-122D10.1 |
| chr15 | 78631512 | 78632194 | 683 | 12 | 1.01E-09 | 0.13 | 0.06 | CRABP1 |
| chr15 | 93362247 | 93362731 | 485 | 2 | 4.80E-10 | -0.15 | -0.14 | NA |
| chr15 | 95869918 | 95870321 | 404 | 5 | 2.23E-08 | -0.12 | -0.06 | CTD-2536I1.1 |
| chr15 | 96596177 | 96596924 | 748 | 6 | 1.16E-07 | -0.17 | -0.10 | NA |
| chr15 | 96865777 | 96867150 | 1374 | 5 | 1.04E-11 | -0.14 | -0.10 | NR2F2-AS1 |
| chr15 | 96886805 | 96888433 | 1629 | 12 | 3.69E-35 | -0.20 | -0.12 | NA |
| chr16 | 10912331 | 10912718 | 388 | 6 | 3.23E-09 | -0.14 | -0.09 | TVP23A |
| chr16 | 70680104 | 70680817 | 714 | 8 | 6.92E-08 | -0.15 | -0.08 | IL34 |
| chr17 | 2324794 | 2325143 | 350 | 5 | 2.31E-09 | -0.14 | -0.11 | METTL16 |
| chr17 | 38333933 | 38334623 | 691 | 8 | 1.17E-08 | -0.11 | -0.04 | RAPGEFL1 |
| chr17 | 46604393 | 46604774 | 382 | 3 | 6.39E-08 | -0.20 | -0.14 | NA |
| chr17 | 59564657 | 59564954 | 298 | 4 | 5.66E-07 | -0.13 | -0.10 | NA |
| chr17 | 60828157 | 60828406 | 250 | 3 | 1.07E-06 | -0.18 | -0.16 | NA |
| chr17 | 79366851 | 79366853 | 3 | 2 | 2.93E-07 | -0.09 | -0.06 | NA |
| chr19 | 14584761 | 14584926 | 166 | 2 | 1.11E-06 | -0.13 | -0.12 | PTGER1 |
| chr19 | 43918465 | 43919039 | 575 | 8 | 7.29E-07 | 0.07 | 0.04 | TEX101 |
| chr2 | 63275509 | 63277327 | 1819 | 28 | 6.99E-14 | -0.16 | -0.05 | OTX1, AC009501.4 |
| chr2 | 88583529 | 88583805 | 277 | 4 | 1.30E-08 | 0.13 | 0.10 | NA |
| chr2 | 1.3E+08 | 1.3E+08 | 631 | 6 | 3.43E-07 | 0.10 | 0.08 | NA |
| chr2 | 1.35E+08 | 1.35E+08 | 767 | 6 | 1.62E-09 | -0.20 | -0.13 | NA |
| chr20 | 22401074 | 22401407 | 334 | 4 | 9.62E-08 | 0.11 | 0.09 | RP5-1004I9.1 |
| chr20 | 33103246 | 33103584 | 339 | 3 | 4.13E-08 | -0.13 | -0.08 | DYNLRB1 |
| chr20 | 62317640 | 62317768 | 129 | 2 | 1.19E-06 | 0.12 | 0.07 | RTEL1, RTEL1-TNFRSF6B |
| chr21 | 36041334 | 36041699 | 366 | 7 | 1.67E-07 | -0.13 | -0.10 | CLIC6 |
| chr21 | 36164119 | 36164167 | 49 | 3 | 1.71E-06 | 0.13 | 0.04 | NA |
| chr22 | 32555255 | 32555310 | 56 | 4 | 7.95E-08 | 0.10 | 0.08 | C22orf42, RP1-90G24.8 |
| chr22 | 36806001 | 36806655 | 655 | 6 | 3.00E-07 | -0.11 | -0.07 | NA |
| chr22 | 45596797 | 45597767 | 971 | 10 | 3.53E-14 | 0.17 | 0.09 | MIR1249, KIAA0930 |
| chr22 | 45899292 | 45899736 | 445 | 3 | 3.48E-07 | -0.10 | -0.09 | FBLN1 |
| chr3 | 350503 | 351003 | 501 | 6 | 2.21E-07 | 0.14 | 0.09 | RPS8P6 |
| chr3 | 24536765 | 24537801 | 1037 | 17 | 1.81E-17 | -0.16 | -0.05 | THRB, THRB-AS1 |
| chr3 | 35785377 | 35786030 | 654 | 6 | 2.79E-07 | 0.10 | 0.07 | MIR128-2 |
| chr3 | 55517496 | 55518441 | 946 | 11 | 2.35E-09 | 0.19 | 0.15 | NA |
| chr3 | 72149324 | 72149807 | 484 | 4 | 2.59E-09 | -0.19 | -0.11 | LINC00877 |
| chr3 | 1.39E+08 | 1.39E+08 | 1203 | 9 | 5.67E-13 | -0.21 | -0.07 | RBP1, RP11-319G6.1 |
| chr3 | 1.83E+08 | 1.83E+08 | 281 | 4 | 1.24E-07 | 0.13 | 0.08 | NA |
| chr3 | 1.86E+08 | 1.86E+08 | 1279 | 9 | 2.83E-20 | -0.19 | -0.11 | NA |
| chr4 | 26030677 | 26030691 | 15 | 2 | 2.27E-06 | -0.05 | -0.04 | NA |
| chr4 | 54975593 | 54976184 | 592 | 5 | 2.58E-07 | -0.07 | -0.06 | NA |
| chr4 | 74486080 | 74486558 | 479 | 10 | 8.09E-09 | -0.11 | -0.06 | RASSF6 |
| chr4 | 1.75E+08 | 1.75E+08 | 208 | 5 | 1.32E-09 | -0.18 | -0.15 | RP11-161D15.3 |
| chr4 | 1.89E+08 | 1.89E+08 | 878 | 8 | 2.71E-11 | -0.14 | -0.10 | NA |
| chr5 | 37834672 | 37836392 | 1721 | 18 | 2.32E-19 | -0.25 | -0.13 | GDNF |
| chr5 | 43017561 | 43018443 | 883 | 6 | 5.66E-09 | -0.14 | -0.09 | CTD-2201E18.3, CTD-2035E11.3 |
| chr5 | 72595685 | 72595958 | 274 | 4 | 1.33E-06 | -0.07 | -0.05 | NA |
| chr5 | 76010444 | 76011698 | 1255 | 11 | 9.32E-17 | -0.13 | -0.06 | F2R, CTD-2384B11.2 |
| chr5 | 92908771 | 92909434 | 664 | 5 | 2.15E-09 | -0.22 | -0.16 | NR2F1-AS1 |
| chr5 | 1.1E+08 | 1.1E+08 | 93 | 2 | 2.12E-06 | -0.14 | -0.12 | TSLP |
| chr5 | 1.41E+08 | 1.41E+08 | 1414 | 10 | 1.90E-14 | -0.13 | -0.07 | PCDHGC4 |
| chr6 | 10883834 | 10884314 | 481 | 6 | 1.69E-07 | -0.12 | -0.08 | GCM2, RP11-637O19.2 |
| chr6 | 25726884 | 25727107 | 224 | 8 | 1.34E-06 | 0.07 | 0.05 | HIST1H2BA, HIST1H2AA |
| chr6 | 28963307 | 28963820 | 514 | 9 | 3.22E-07 | 0.07 | 0.04 | NA |
| chr6 | 29690766 | 29691250 | 485 | 9 | 6.32E-11 | 0.25 | 0.09 | HLA-F, HCG4P11 |
| chr6 | 30104440 | 30104916 | 477 | 9 | 1.09E-06 | 0.08 | 0.05 | TRIM40 |
| chr6 | 30113696 | 30114039 | 344 | 8 | 5.76E-08 | 0.11 | 0.06 | NA |
| chr6 | 30127760 | 30128775 | 1016 | 15 | 4.77E-07 | 0.08 | 0.04 | TRIM10 |
| chr6 | 31238751 | 31239411 | 661 | 7 | 1.53E-09 | 0.12 | 0.04 | HLA-C |
| chr6 | 31650735 | 31651362 | 628 | 21 | 9.37E-16 | -0.15 | -0.07 | LY6G5C |
| chr6 | 32117558 | 32119041 | 1484 | 20 | 1.64E-17 | -0.14 | -0.05 | PRRT1 |
| chr6 | 41528395 | 41528623 | 229 | 4 | 2.16E-06 | -0.16 | -0.14 | NA |
| chr6 | 46047619 | 46047991 | 373 | 2 | 1.78E-10 | 0.09 | 0.07 | CLIC5 |
| chr6 | 1.34E+08 | 1.34E+08 | 1012 | 12 | 1.75E-10 | -0.12 | -0.08 | TCF21, RP3-323P13.2 |
| chr7 | 19158349 | 19158954 | 606 | 10 | 4.64E-08 | -0.15 | -0.07 | TWIST1, AC003986.7 |
| chr7 | 26192756 | 26193109 | 354 | 4 | 1.42E-07 | -0.16 | -0.11 | NFE2L3 |
| chr7 | 27142799 | 27144302 | 1504 | 15 | 2.36E-18 | -0.16 | -0.08 | HOXA2 |
| **chr7** | **27161624** | **27162768** | **1145** | **6** | **4.78E-09** | **-0.12** | **-0.11** | **HOXA-AS2** |
| chr7 | 27168609 | 27168780 | 172 | 3 | 1.93E-06 | -0.10 | -0.08 | HOXA3, HOXA4, HOXA-AS3 |
| chr7 | 27183369 | 27185512 | 2144 | 46 | 3.69E-35 | -0.20 | -0.10 | HOXA5, HOXA6, HOXA-AS3 |
| chr7 | 27187102 | 27187670 | 569 | 9 | 8.53E-07 | -0.13 | -0.08 | HOXA6, HOXA-AS3 |
| chr7 | 27204005 | 27205658 | 1654 | 20 | 5.37E-11 | -0.16 | -0.05 | HOXA9 |
| chr7 | 50633725 | 50633773 | 49 | 2 | 1.09E-06 | 0.13 | 0.10 | DDC |
| chr7 | 55224743 | 55225183 | 441 | 7 | 1.47E-07 | -0.21 | -0.17 | NA |
| chr7 | 99680278 | 99680287 | 10 | 3 | 5.36E-08 | -0.06 | -0.06 | ZNF3 |
| chr7 | 1.31E+08 | 1.31E+08 | 28 | 2 | 1.62E-07 | 0.12 | 0.11 | NA |
| chr7 | 1.51E+08 | 1.51E+08 | 253 | 5 | 7.18E-07 | -0.12 | -0.08 | CRYGN |
| chr8 | 23584042 | 23584760 | 719 | 6 | 2.47E-07 | -0.10 | -0.04 | NA |
| chr8 | 37378276 | 37378424 | 149 | 3 | 7.75E-07 | -0.18 | -0.14 | NA |
| chr8 | 58191386 | 58192065 | 680 | 8 | 5.24E-07 | 0.07 | 0.05 | LINC00588 |
| chr8 | 77617694 | 77618401 | 708 | 7 | 1.90E-08 | 0.09 | 0.07 | ZFHX4 |
| chr8 | 1.04E+08 | 1.04E+08 | 1002 | 10 | 2.62E-09 | -0.13 | -0.06 | BAALC, C8orf56 |
| chr8 | 1.43E+08 | 1.43E+08 | 560 | 4 | 1.58E-09 | 0.11 | 0.08 | NA |
| chr9 | 90113998 | 90114156 | 159 | 3 | 4.27E-07 | -0.13 | -0.11 | DAPK1 |
| chr9 | 1.35E+08 | 1.35E+08 | 461 | 3 | 7.58E-07 | -0.09 | -0.05 | NA |
