# Supplementary material for: TWIST1 DNA methylation is a cell marker of airway and parenchymal lung fibroblasts that are differentially methylated in asthma
Source: Clin Epigenetics. 2020 Oct 2;12:145. doi: 10.1186/s13148-020-00931-4 (PMC7531162; doi:10.1186/s13148-020-00931-4)
Supplement: Supplementary file 2 — Additional file 2. Supplementary Tables 1-8 [file 13148_2020_931_MOESM2_ESM.zip › ST7.docx]

**Supplementary Table 7: Summary of the 78 CpGs verified as differentially methylated between airway and parenchymal fibroblasts in paired samples**

| **CpG Site** | **Chromosome** | **Position** | **Delta Beta** | **Bonferroni p value** |
| --- | --- | --- | --- | --- |
| cg01135464 | chr17 | 48971001 | 0.80 | 0.000020 |
| cg08777316 | chr5 | 158531511 | -0.73 | 0.000145 |
| cg10186131 | chr15 | 96887560 | -0.70 | 0.000273 |
| cg12479878 | chr1 | 221054825 | -0.93 | 0.000311 |
| cg20454002 | chr1 | 221054365 | -0.72 | 0.000350 |
| cg22840361 | chr15 | 96887919 | -0.67 | 0.000369 |
| cg26104083 | chr6 | 1606394 | -0.76 | 0.000377 |
| cg02326806 | chr15 | 96887461 | -0.78 | 0.000401 |
| cg13972986 | chr7 | 4857070 | 0.51 | 0.000479 |
| cg00032912 | chr15 | 96887529 | -0.71 | 0.000530 |
| cg22698272 | chr1 | 221054615 | -0.83 | 0.000700 |
| cg16935039 | chr6 | 1607507 | -0.65 | 0.000719 |
| cg19306970 | chr1 | 221055097 | -0.82 | 0.000728 |
| cg18731398 | chr3 | 123414733 | -0.66 | 0.000739 |
| cg19243409 | chr7 | 127963545 | -0.64 | 0.000983 |
| cg19598567 | chr5 | 141931207 | -0.63 | 0.001358 |
| cg05289920 | chr15 | 96886805 | -0.74 | 0.001505 |
| cg10624122 | chr7 | 19158747 | -0.63 | 0.001649 |
| cg09251600 | chr1 | 179781780 | -0.64 | 0.002039 |
| cg02242345 | chr16 | 66938788 | -0.57 | 0.002135 |
| cg20992181 | chr11 | 69706285 | -0.59 | 0.002489 |
| cg25890168 | chr18 | 19262819 | -0.58 | 0.002520 |
| cg09088153 | chr15 | 96890598 | -0.85 | 0.002568 |
| cg03607680 | chr1 | 48450020 | -0.66 | 0.002610 |
| cg01894498 | chr14 | 61655848 | -0.57 | 0.002830 |
| cg07665222 | chr11 | 125552186 | -0.72 | 0.003007 |
| cg25799109 | chr3 | 57102900 | 0.59 | 0.003432 |
| cg06187770 | chr6 | 85484863 | -0.68 | 0.003638 |
| cg23817096 | chr6 | 1620687 | -0.52 | 0.003732 |
| cg09930712 | chr7 | 28838086 | 0.73 | 0.003737 |
| cg15100426 | chr2 | 219187432 | -0.55 | 0.004043 |
| cg00685614 | chr16 | 89118838 | 0.67 | 0.004799 |
| cg21159940 | chr7 | 28838234 | 0.63 | 0.005244 |
| cg15193215 | chr6 | 1841153 | 0.59 | 0.005766 |
| cg07116712 | chr15 | 96887959 | -0.71 | 0.005793 |
| cg06357305 | chr20 | 48183673 | 0.54 | 0.006278 |
| cg01514538 | chr1 | 2980380 | -0.68 | 0.006561 |
| cg08263708 | chr6 | 85476373 | -0.54 | 0.006600 |
| cg26122286 | chr13 | 101196636 | -0.55 | 0.007170 |
| cg22178798 | chr15 | 96890626 | -0.87 | 0.008252 |
| cg04484550 | chr1 | 2980163 | -0.74 | 0.008290 |
| cg09074856 | chr1 | 2979817 | -0.63 | 0.009382 |
| cg10151901 | chr15 | 96886519 | -0.57 | 0.009961 |
| cg14030882 | chr14 | 61655919 | -0.53 | 0.010151 |
| cg03957885 | chr10 | 89855267 | -0.58 | 0.010723 |
| cg09452082 | chr15 | 96886018 | -0.74 | 0.010902 |
| cg23008352 | chr13 | 110951025 | -0.54 | 0.011132 |
| cg05766064 | chr18 | 19262576 | -0.58 | 0.011646 |
| cg15590989 | chr4 | 183062460 | -0.52 | 0.012590 |
| cg04572930 | chr7 | 4754834 | -0.79 | 0.012875 |
| cg14391419 | chr7 | 19158647 | -0.61 | 0.013309 |
| cg17140992 | chr15 | 96887137 | -0.57 | 0.014519 |
| cg17454263 | chr15 | 96886397 | -0.52 | 0.015340 |
| cg03554749 | chr4 | 85405132 | -0.51 | 0.016819 |
| cg09990962 | chr1 | 3164431 | -0.71 | 0.016839 |
| cg17001566 | chr1 | 2990490 | -0.61 | 0.020812 |
| cg26540315 | chr11 | 69264657 | -0.65 | 0.024921 |
| cg05740106 | chr6 | 169273346 | 0.58 | 0.025391 |
| cg06096901 | chr17 | 78894393 | 0.52 | 0.025667 |
| cg23095243 | chr14 | 32435490 | -0.56 | 0.027652 |
| cg24939838 | chr1 | 3164390 | -0.71 | 0.029295 |
| cg07319605 | chr2 | 114474155 | -0.50 | 0.029654 |
| cg06206801 | chr18 | 24131379 | -0.53 | 0.031851 |
| cg23543318 | chr4 | 1195845 | -0.64 | 0.032967 |
| cg26219674 | chr1 | 154680613 | -0.54 | 0.033179 |
| cg22460896 | chr15 | 96888024 | -0.58 | 0.033421 |
| cg03730533 | chr7 | 26897685 | -0.50 | 0.034140 |
| cg16174681 | chr5 | 108220453 | -0.67 | 0.037634 |
| cg18261462 | chr17 | 46507705 | 0.58 | 0.037900 |
| cg16872841 | chr13 | 110993558 | -0.67 | 0.039023 |
| cg06122871 | chr6 | 85476210 | -0.51 | 0.039687 |
| cg22712983 | chr2 | 219187374 | -0.65 | 0.041305 |
| cg12140851 | chr7 | 26897612 | -0.53 | 0.042542 |
| cg00033666 | chr15 | 96887043 | -0.55 | 0.044300 |
| cg14615927 | chr2 | 240259213 | -0.66 | 0.044537 |
| cg18877525 | chr1 | 244303292 | -0.51 | 0.046079 |
| cg26762347 | chr1 | 2990031 | -0.65 | 0.046335 |
| cg25131079 | chr5 | 178712051 | -0.51 | 0.046520 |
