# Supplementary material for: TWIST1 DNA methylation is a cell marker of airway and parenchymal lung fibroblasts that are differentially methylated in asthma
Source: Clin Epigenetics. 2020 Oct 2;12:145. doi: 10.1186/s13148-020-00931-4 (PMC7531162; doi:10.1186/s13148-020-00931-4)
Supplement: Supplementary file 2 — Additional file 2. Supplementary Tables 1-8 [file 13148_2020_931_MOESM2_ESM.zip › ST8.docx]

**Supplementary Table 8: Summary of the 14 DNA regions validated as differentially methylated between airway and parenchymal fibroblasts by DMRcate analysis of matched cell samples**

| Chr | Start | End | Width | No. CpGs | Min. FDR | Max. Beta diff. | Mean Beta diff. | Gene |
| --- | --- | --- | --- | --- | --- | --- | --- | --- |
| chr1 | 2979311 | 2980546 | 1236 | 6 | 9.99E-37 | -0.48 | -0.43 | LINC00982 |
| chr1 | 221054365 | 221055097 | 733 | 4 | 8.14E-28 | -0.56 | -0.54 | HLX, HLA-AS1 |
| chr14 | 61655848 | 61655919 | 72 | 3 | 1.03E-40 | -0.57 | -0.50 | PRKCH |
| chr15 | 96885119 | 96888024 | 2906 | 13 | 2.63E-117 | -0.66 | -0.48 | NA |
| chr3 | 128210888 | 128212273 | 1386 | 6 | 7.27E-09 | -0.24 | -0.19 | GATA2 |
| chr3 | 169377725 | 169377946 | 222 | 3 | 2.27E-36 | 0.64 | 0.59 | NA |
| chr4 | 85422453 | 85424469 | 2017 | 6 | 3.80E-26 | -0.38 | -0.28 | NA |
| chr4 | 85402397 | 85403409 | 1013 | 4 | 7.78E-21 | -0.38 | -0.30 | NA |
| chr6 | 85476210 | 85476921 | 712 | 3 | 1.50E-32 | -0.53 | -0.47 | TBX18 |
| chr7 | 19158349 | 19158747 | 399 | 6 | 1.16E-66 | -0.58 | -0.53 | TWIST1 |
| chr7 | 26897597 | 26897714 | 118 | 4 | 7.65E-41 | -0.53 | -0.48 | SKAP2 |
| chr7 | 1961785 | 1961968 | 184 | 3 | 1.67E-07 | 0.24 | 0.22 | NA |
| chr7 | 95546508 | 95546556 | 49 | 3 | 1.71E-28 | -0.53 | -0.44 | NA |
| chr8 | 6419483 | 6420242 | 760 | 3 | 2.42E-20 | -0.49 | -0.37 | ANGPT2 |
